# Religiosity is associated with greater size, kin density, and geographic dispersal of women’s social networks in Bangladesh

**DOI:** 10.1038/s41598-022-22972-w

**Published:** 2022-11-05

**Authors:** R. Lynch, S. Schaffnit, R. Sear, R. Sosis, J. Shaver, N. Alam, T. Blumenfield, S. M. Mattison, M. Shenk

**Affiliations:** The Pennsylvania State University, State College, USA

**Keywords:** Anthropology, Ecological networks, Evolutionary ecology

## Abstract

Human social relationships, often grounded in kinship, are being fundamentally altered by globalization as integration into geographically distant markets disrupts traditional kin based social networks. Religion plays a significant role in regulating social networks and may both stabilize extant networks as well as create new ones in ways that are under-recognized during the process of market integration. Here we use a detailed survey assessing the social networks of women in rural Bangladesh to examine whether religiosity preserves bonds among kin or broadens social networks to include fellow practitioners, thereby replacing genetic kin with unrelated co-religionists. Results show that the social networks of more religious women are larger and contain more kin but not more non-kin. More religious women’s networks are also more geographically diffuse and differ from those of less religious women by providing more emotional support, but not helping more with childcare or offering more financial assistance. Overall, these results suggest that in some areas experiencing rapid social, economic, and demographic change, religion, in certain contexts, may not serve to broaden social networks to include non-kin, but may rather help to strengthen ties between relatives and promote family cohesion.

## Introduction

Religion is expected to influence social relationships^1^, but how religious beliefs and practices impact relationships between kin and non-kin is largely unknown. This is particularly true in many lower and middle-income countries where religion is often nearly universal and little, or no evidence of secularization exists. In other words, how does an individual’s religious beliefs and practices affect their kin and non-kin social networks, and how is this relationship affected by one’s level of market integration? These questions are of particular importance in societies undergoing rapid integration into a market economy where urbanization, major demographic changes (i.e., decline in fertility) and changing occupations and ways of life are fundamentally reshaping families and social networks.

Kinship has provided a framework for human social life and has been crucial to our species' success^2–5^. Traditional kinship systems, however, are often disrupted by the transition from subsistence to market-based economies^6,7^, as institutions and structures previously organized around families increasingly depend on ties among unrelated individuals that are frequently organized around collective identities^8,9^. Globalization and the decline of complex family living arrangements^10–13^ have further accelerated this process, fundamentally changing social relationships^7,14^ by reducing the kin-density of social networks^15,16^ and support from relatives^17^.

Meanwhile, many researchers have argued that religion has played an important role in cultural evolution by promoting cooperation and enabling larger and more complex human societies to evolve^18^. In particular, the intensity of an individual’s religious beliefs and practices is expected to have an important effect on one’s social relationships^19^. Indeed, *religiosity*—an individual’s degree of devotion to their religion—has been associated with more satisfying relationships and spending time with both relatives^20–22^ and non-relatives^23,24^ as religious traditions and beliefs can affect social behavior by broadening who is seen as a member of one’s in-group^25,26^. Religion may therefore play an important role in the global shift from more kin based ‘intensive’ social networks to the less kin-based ‘extensive’ social networks that are typically associated with market integration^7,27^.

Religious practices are not only expected to reshape individual social networks, however, they are also likely to impact social capital—the level of trust, coordination and cooperation in a society^28,29^—more broadly. Deteriorating social contact resulting from modernization^30^, increasing geographic mobility^10^, and the rise of social media^31^ can all conspire to undermine traditional social relationships^32,33^. Although social capital is generally seen as important for the well-being of both individuals and populations, researchers frequently distinguish between strong ties within closed groups—*bonding social capital*— and weak ties linking different groups—*bridging social capital*^34,35^, and these types of connections can have very different effects. Relationships among kin, which are seen as *bonding* social networks, have, for example, been associated with higher fertility, while more *bridging* ties, such as those between people from different ethnic backgrounds, have been shown to improve economic outcomes^36^. Although different religious traditions in different contexts can have disparate effects on social capital^37^, religions in general are thought to affect social relationships by either^33,38^ (1) increasing bonding capital by strengthening relationships amongst kin, or (2) increasing bridging capital by helping to expand social networks to include non-kin.

There is good evidence for the first proposition that stronger religious beliefs result i closer relationships with kin and increase family cohesion^21,22,39,40^. Indeed religious practices themselves may foster tighter bonds within families^41,42^ and help to sanctify (i.e., provide a spiritual character and significance to) relationships among kin^43,44^. For example, studies have reported positive associations between religious beliefs and father-child bonds^45^, marital satisfaction^46^, and relationships among siblings^47^. These patterns of increased within-family cooperation may even help to explain why more religious people in post-demographic transition societies frequently have more children than their less religious counterparts^48–50^. More broadly, this perspective suggests that religion may buffer any effects of modernization on eroding kin networks. In this interpretation, religion acts to counteract the potentially disruptive impacts of economic modernization on bonds between family members.

There is also support for the second contention that religion expands social networks to include more non-relatives. Here religion is seen to replace kin-based networks with ties to unrelated co-religionists^18,51,52^. Durkheim^53^, for instance, argued that religious rituals serve to bond unrelated group members and increase group cohesion, while Rappaport^54^ suggested that these rituals increase social solidarity by signaling adherence to a moral code, which, in turn builds trust and facilitates cooperation among unrelated religious group members. Evidence showing that religious individuals have closer relationships with non-relatives than non-religious individuals^55,56^ supports this hypothesis, but does not tell us whether this happens at the expense of kin-based networks. However, market integration and modern labor markets increase the incentives for people to move further away from their families in search of employment^57^, which might reduce interactions with relatives and increase interactions with non-relatives^57,58^.

These two pathways are not mutually exclusive, however. Religion could strengthen relationships among kin while also expanding social networks to include more non-kin thereby leading to an overall increase in the size of people’s social networks. It is also possible that religious beliefs have no effect on the number of relationships people have with relatives or non-relatives, but rather affect the character of these relationships (e.g., the type of support received). These questions remain unresolved because the role that religion and religiosity play in the structure of social networks and systems of social support has not been well studied. Moreover, most of the research that has been done has been conducted in post-industrial Western societies like the United States where there are a significant number of religiously unaffiliated people. This is a problem because secularization generally occurs late in the process of economic development and in much of the world rapid economic development has thus far occurred without any coincident decline in religious practice^59^. This heavy focus on societies late in the transition may therefore obscure our understanding of the role that religion plays earlier in the process of market integration where the secular organizations that often come to fulfill some of the functions formerly filled by religious institutions may not (yet) exist^60^.

Here we investigate how religiosity impacts the size, kin composition, and character of women’s social networks in rural Bangladesh, an area undergoing rapid market integration. We seek to answer two related questions: Does religion strengthen and broaden ties among relatives, and/or does it broaden social networks by increasing the number of ties between non-kin? We are able to directly test the first question, and this analysis sheds important light on the second question by assessing relationships among non-kin. We hypothesize that religiosity will partially buffer the effects of market integration and the geographic diffusion of kin networks by helping to preserve social relationships among relatives. We make the following six predictions (the first 5 were pre-registered in^61^: (**P1a**) Higher religiosity will be positively associated with larger overall social networks, which will be primarily driven by (**P1b**) more religious women having more relatives in their networks; (**P2**) Higher religiosity will be associated with the geographic proximity of kin (i.e., the relatives of more religious women will live nearer); (**P3a**) Higher religiosity will be positively associated with more emotional support from kin (i.e., more frequent conversations), (**P3b**) more alloparenting support and care from kin, and (**P3c**) more financial help from kin (not pre-registered).

## Methods

Hypotheses and predictions were pre-registered, and time stamped on the Open Science Framework website. All R code for data, models and construction of figures is publicly available on Github. This study was approved by the Ethical Review Committee of icddr,b and the Institutional Review Board at Pennsylvania State University. Informed consent was obtained from all participants and all aspects of the study were performed in accordance with guidelines and regulations provided by icddr,b and Penn State.

### Sample and study population

Data for this study were drawn from a 2018 survey of 766 women in Matlab, Bangladesh. Women were eligible for inclusion if they were between the ages of 20 and 65 in 2010 when the original wave of data were collected (N = 944), and if they were still living in the study area in 2018. Participants were randomly sampled from an existing population register in 2010 with equal samples drawn from ages 20–34, 35–49, and 50–65 to avoid oversampling of younger women in this growing population. The 2018 data used here included additional information on participants’ religious practices and extensive information on various aspects of market integration, social network composition (including kinship status of all social network members), and types of support received.

Data were collected in the Matlab Health and Demographic Surveillance System (HDSS) site run by the International Centre for Diarrhoeal Disease Research, Bangladesh (icddr,b)^62^. The economy of the region centers on intensive agriculture, with rice as the staple crop, alongside fishing^62^. Although a large proportion of inhabitants participate in agriculture, many do not own land themselves, and the majority of households also participate in wage labor. Household income is often generated from multiple sources which may include farming, fishing, day labor, handicraft production, small businesses, salaried work, and remittances from family members working in cities or abroad^63^. Average annual household income was estimated at $1,584 in 2010 US dollars^64^. While 30% of the population has never attended school^63^, education has become increasingly available, and some residents now have salaried jobs that are education-based. Education has also become more acceptable and common for women^65^. Labor migration, primarily by men, and remittances have become increasingly important for the economy of Matlab^65,66^. These economic changes are linked to both push and pull factors, including decreasing land ownership due to a rising population^67^ and increasing access to national and international markets for labor and goods^66,68^.

Members of one or more extended patrilocal families often live near each other in a neighborhood called a *bari* which can vary from a few to several dozen houses, though extended families increasingly live separately in a town or distant bari driven by local occupational and educational opportunities. Women often practice a limited form of seclusion and spend much of their time in the bari engaged in agricultural processing work, cooking, and childcare^69^; less than 6% of women work outside the home, though some women may make handicrafts or raise fowl. Members of the same bari generally cooperate in childcare, and in the loaning of household items and small amounts of money. Families keep in touch with each other via mobile phone (an emerging trend over the last decade) and frequent visiting (a longstanding custom in the region).

### Religiosity

Bangladesh is a highly religious country, with fewer than 0.2% of the population reporting that they are not religious and more than 85% considering religion to be an important part of their lives^70^. Residents of Matlab are primarily Muslim—89% in our sample—with a minority population of 11% Hindus^63^. Most areas have at least one mosque (*masjid*) or temple (*mandir*), with large institutions in cities and smaller institutions in villages and local *bari*. Many mosques and temples organize religious events for the community, but for many people, institutionally focused practice is less important than practice in the home or neighborhood. While women generally do not work outside of their home or bari, women are active practitioners of religion in both Islam and Hinduism and have important roles in home practice and local community practice (often among other women). Furthermore, while they do not generally have roles as formal religious teachers, they play a key role in conveying religious ideals and practices to children in domestic settings.

Most of the women in our sample received their religious education from religious teachers (88%) or family members (24%). Most Muslims in Matlab pray five times a day; men often pray in a communal setting at the local mosque while women typically pray at home or in a private prayer room. Many Muslim children learn to read and/or recite the Quran in Arabic (59%) and both women and men form groups to read religious texts (the Quran and Hadith) during Ramadan or at other times. Families who can afford it send older men and women to Mecca on hajj (6%) and less wealthy families participate in religious pilgrimages known as umrah (5%) often as part of a local group. Muslims also engage in fasting at Ramadan, perform acts of charity for others, and may signal greater religious engagement through choice of clothing, naming of children, or other socially visible behaviors.

Hindus perform *puja* (worship) at shrines within the household or bari, and at temples in the village or larger region. A few (mainly pandits, i.e., priests) may learn to read the Gita or other texts in Sanskrit (2%), some read translated versions in Bangla (34%), and most learn teachings from family members through oral traditions. Most families and women participate in religious festivals either locally—within the home or bari—or at temples and pilgrimage sites. Wealthier or more pious women may go on pilgrimage to important sites in India (6%), but more commonly women may participate in large religious gatherings or pilgrimages to temples or holy places in Bangladesh (55%), often with kin and/or other community members. Religious devotion may also be shown through dress, frequency of puja or prayer, and acts of charity.

The strength of beliefs and participation in rituals varies across Muslim and Hindu individuals and families in the study area, providing significant variation in religiosity. For this paper we use two measures of religiosity. First, we construct a composite measure of *religious knowledge and participation*, a scaled variable assigning values for survey responses suggesting greater knowledge and proficiency in the respondent’s religion, including amount and formality of religious education, ability to read and/or recite religious texts, and participation and frequency of religious pilgrimages. This scale ranged from 1 (lowest) to 20 (highest) with a median score of 3.0. These specific measures of religious knowledge and behavior only capture particular dimensions of religiosity, however, and thus may not effectively capture religiosity overall, so we also asked respondents to rate their own *relative religiosity* with the question ‘Compared to other families in this bari/village how religious is your family?’ (more, less, or same). This question allowed participants to self-report their religiosity in the most locally appropriate way, resulting in an ethnographically valid measure that encompasses dimensions of religiosity that are not easy to directly measure. This self-report measure also avoids problems with reductionism and imposing an etic perspective that are inherent in a specific list of dimensions, and thus more precisely gets at the central question in this paper—i.e., whether having higher or lower religiosity compared to others in the community (as understood by members of that community) is associated with variation in the structure of social networks. This variable shows significant variation across respondents, with 11% reporting being less religious than others, 53% reporting that they are the same as others, and 36% reporting that they are more religious that others (see Table S1). This distribution does not suggest overreporting of the more religious category, which appears as often as one would expect it to by chance, though the distribution does suggest that respondents may be lumping responses in the middle category and avoiding the lowest category, possibly as a form of impression management. However, given the high level of religious practice in this area, it may also be true that most women fall into a middle category of moderately religious. Although using this simple relative measure of religiosity we may miss details on particular dimensions of religious belief and practice^26^, it is also true that any typology of religious behaviors (including our scale of religious knowledge and participation) is likely to miss key elements of practice, suggesting the utility of a holistic and ethnographically meaningful approach.

All questions were extensively pre-tested and revised before data collection with input from local residents and research assistants. Moreover, both pre-testing and focus discussion groups revealed that participants clearly understood the questions about religious knowledge and participation and found the concept of being more or less religious than others clearly interpretable in the local context—indeed both the specific dimensions of religious knowledge measured, and the relative religiosity question were suggested to us by local research assistants. Answers to open-ended focus group questions about how individuals are able to determine the religiosity of someone in their community were centered around observing how much they pray, their use of language (e.g., avoiding bad words), using the proper religious greeting when they meet someone (e.g., Salaam amongst Muslims), and observance of norms of modest dress (e.g., Muslim women covering their hair or veiling when traveling outside of their home community).

### Social networks

**Figure 1:**
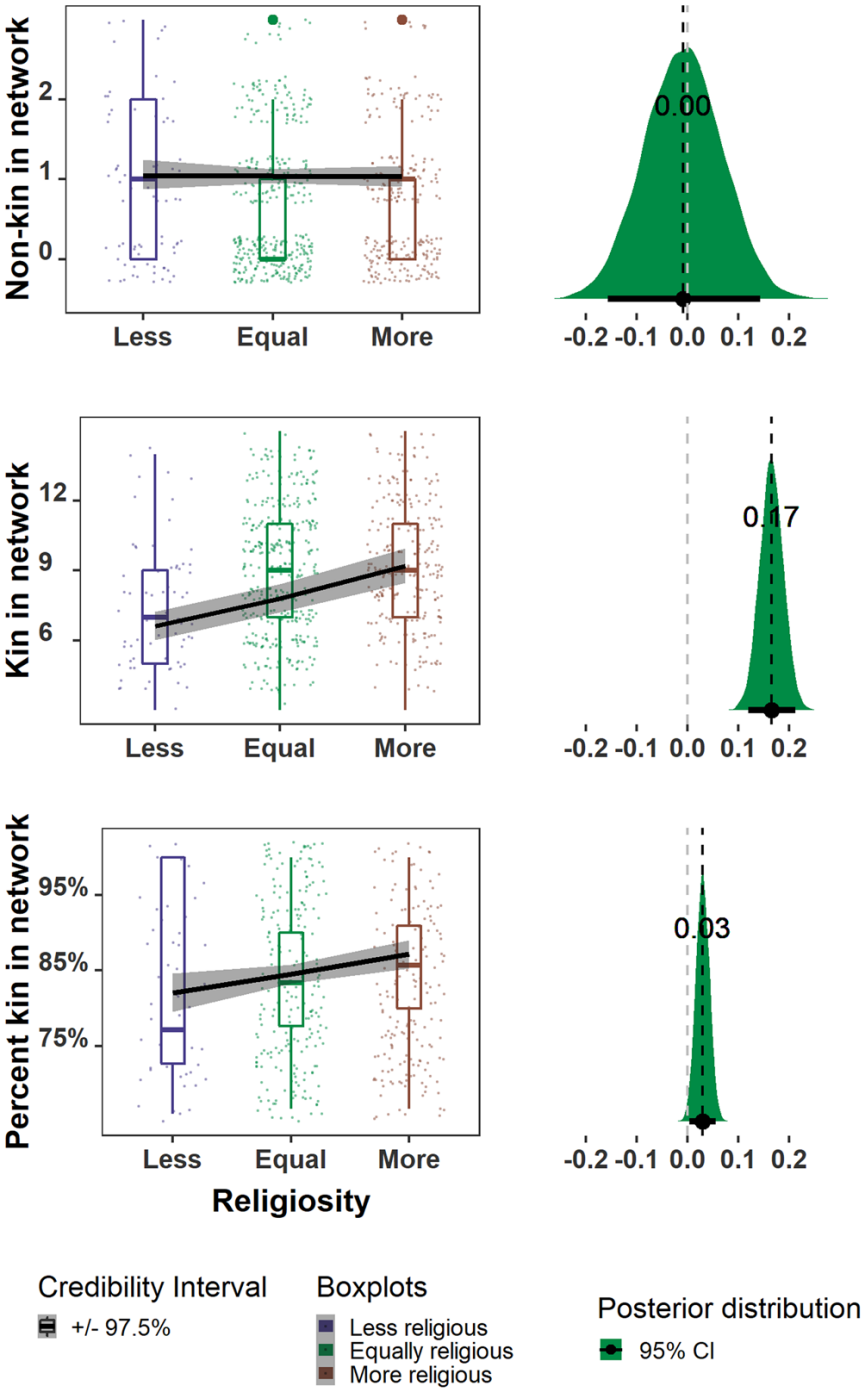
Social network size and composition (Y-axis) across increasing degrees of religiosity (X-axis). More religious women have larger networks overall (top row), a similar number of non-kin in their networks (2nd row), more family members in their networks (3rd row), and a higher percentage of family members in their networks (bottom row). Raw data points and error bars represent 97.5 percent credibility intervals for each category of increasing religiosity (left side) and the density interval for the respective posterior distributions and mean parameter estimates for religiosity for each model (right).

Social network members were listed by participants in response to a series of questions and include anyone whom the respondent identified as someone who (1) helps them or they help, (2) they spend significant time with, (3) serves (or for whom they serve) as a wealthy or prestigious social connection, (4) helps them (or whom they help) with childcare or housework, (5) they would ask (or be asked by) to loan them money, (6) they discuss important matters and ask advice from (or give advice to) and, (7) they discuss important matters with (see **SM attachment**: 2018 Household Questionnaire)*.* Participants were asked to free list individuals in response to each question and were prompted with “who else?” and/or “is there anyone else” until they no longer continued listing individuals. We calculate network size as the total number of distinct individuals (i.e., we removed duplicates) listed by participants in response to all social network questions. We also examine the number of individuals listed in response to particular questions. Individuals were categorized as *helping financially* if they were listed as people who the respondent would either loan or borrow money from, as *helping with childcare* if they helped them or they helped with childcare or housework, and as having *provided emotional support* if they were listed as people with whom they discuss important matters and those with whom they spend significant time. For each member of their social network, participants were asked whether that person was a relative or not and we again calculated the number of distinct individuals listed who were either relatives or non-relatives. Given the high density of settlement in rural Bangladesh, individuals would generally have many social contacts (both kin and non-kin) to nominate, so we do not expect network size in this location to be limited by local availability of potential partners (Fig. 1).

### Market integration

Three indices of market integration (see **SM text: Market Integration)** were developed using Exploratory Factor Analysis (EFA) in an attempt to capture a latent construct measuring the degree to which each household was both dependent on supply chains^71^ and integrated into global markets^72^. In all, we began with 213 variables from the 2018 household survey on market integration (see **SM attachment**: *2018 Household Questionnaire*)—152 measuring the assets and hence overall wealth of households, 24 measuring occupations, and 37 additional variables assessing other aspects of market integration—to generate three orthogonal indices of *market integration.* In the end, 27 variables, including composite measures compiled from a number of underlying variables, were included in the resulting three factors. We interpret the three new latent variables generated by this process as indexing geographic proximity (MI1), economic capital (MI2), and human capital (MI3) (see Supplementary materials: Table S4).

### Geographic distance

**Figure 2:**
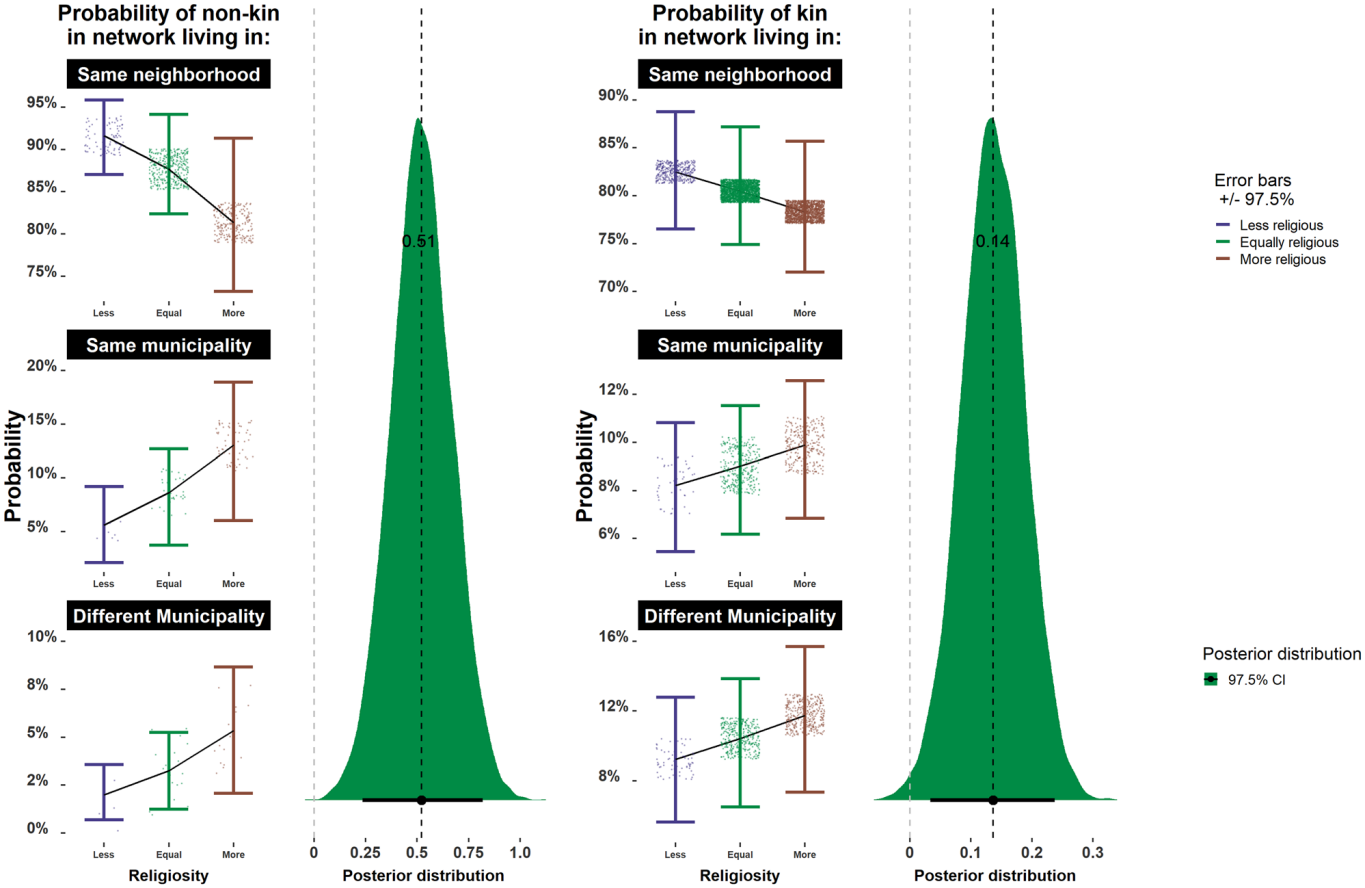
The relationship between religiosity (X-axis) and the geographic distance of non-kin (left) and kin (right). More religious women have more geographically diffuse social networks, especially with non-kin. There is a lower probability that a given member of a woman’s social network will live in the same neighborhood if they are more religious (top row). At the same time, more religious women are also more likely to have both kin and non-kin living outside of their neighborhood (i.e., in the same or different municipality) (bottom two rows). Raw data points and error bars represent 97.5 percent credibility intervals for each category of increasing religiosity (left side) and the density interval for the respective posterior distributions and mean parameter estimates for religiosity for each model (right).

Geographic distance to relatives and non-relatives (see Table 1 and Fig. 2) was coded into an ordinal response variable based on geographic proximity as reported by participants. The location of all individuals listed in social networks were also recorded and categorized as 1) being a member of the same household or a near neighbor (i.e., living in the same or a nearby bari), 2) living somewhere else in Matlab, or 3) living outside of Matlab either in Bangladesh or abroad. It is important to note that this measure is distinct from the ‘geographic proximity’ measure we generated through EFA that was used to assess a dimension of market integration, based on travel times to various locations such as small or large markets, town schools or hospitals (see SM text: Market Integration).

**Table 1:** Effect of religiosity on multiple dependent variables. Parameter estimate [$\upbeta$], 97.5% Bayesian Credibility Intervals (CI) and Rhat values which provides information on the convergence of the Markov chains with values near 1 suggesting good mixing (cf., Gelman and Rubin, 1992).

	All parameter estimates are for relative religiosity			
Dependent variable	Estimate [$\upbeta$ $\upbeta$]	CI 2.5%	CI 97.5%	Rhat
Geographic distance from non-relatives*	0.51	0.28	0.75	1.01
Geographic distance from relatives*	0.14	0.07	0.21	1.03
Total Network Size*	0.12	0.08	0.15	1.00
Non-relatives in network	0.01	−0.13	0.15	0.99
Relatives in network*	0.13	0.09	0.17	1.00
Percent relatives in network*	0.03	0.01	0.05	1.00
Non-relatives who give financial support	−0.20	−0.48	0.08	1.00
Relatives who provide financial support	−0.01	−0.06	0.05	1.00
Percent relatives who provide financial support	0.04	−0.08	0.15	1.00
Non-relatives who help with childcare*	−1.30	−1.94	−0.71	1.00
Relatives who help with childcare	−0.01	−0.06	0.04	1.02
Percent relatives who help with childcare	0.05	−0.05	0.15	1.00
Non-relatives who provide emotional support	−0.09	−0.48	0.31	1.00
Relatives who provide emotional support*	0.19	0.15	0.23	1.00
Percent relatives who provide emotional support	0.04	−0.06	0.15	1.00

CI estimates that do not overlap with 0 have been marked with an asterisk although we recognize that there is nothing special about this designation.

*Result s controlled for the age of the respondent, three indices of market integration (see Methods: market integration), sex, religion, religious knowledge and the number of children in the household.

### Statistical analysis and models

We used Bayesian generalized linear mixed-effects regression models (GLMM) using the brms package^73^ in R Studio 4.0.3^74^ to model the social networks of 767 households in Matlab, Bangladesh. All R code for data, models and construction of figures is publicly available on Github. We ran models for 15 outcome variables related to the size, kin density (number of relatives, non-relatives and percentage of relatives), and geographic proximity of participants’ social networks across various dimensions of support (see Table 1 for all dependent variables).

Predictors and covariates were identical across all models (see **SM**: Table S1 for descriptive statistics and frequencies). Our key predictor was *relative religiosity* (more, less, same). After preliminary analyses, relative religiosity was selected as our main predictor of interest because it was less influenced by socioeconomic status than the religious knowledge and participation scale, which was positively correlated with two of our measures of market integration—human capital: r = 0.28 and economic capital: r = 0.18. We retain the religious knowledge and participation scale as a control in our models; however, none of the models substantively changes when this variable is removed, suggesting that our results are primarily driven by relative religiosity.

Our three dimensions assessing market integration—geographic proximity to markets, economic capital, and human capital were also included as control variables in all models (see Market Integration above) (see Github (^75^ Kin_density_DV.R for R code). We also controlled for the respondent’s number of children and number of siblings; this was done to ensure that we were not simply capturing the effect of large family or sibship sizes in the current or previous generation but instead targeting the relationship between relative religiosity and social network size with the effect of family size removed. These variables were all entered as fixed effects. In addition, religion (Muslim = 1, Hindu = 0) was entered as a random effect in all models, allowing for each religion to have a different mean while still estimating the global mean. This method provides feedback across clusters and improves out of sample predictions through shrinkage (i.e. allowing the data to learn across subgroups) and partial pooling to improve group estimates, particularly those with smaller sample sizes (in this case Hindus)^76^. Interactions were also run between all three market integration factors and relative religiosity to capture the possibility that relative religiosity exerts different effects on social networks at varying levels of market integration.

Models were fit using a Hamiltonian Monte Carlo algorithm, implemented in version 2.12 of Stan^73^, to draw samples from the posterior distribution. We assessed convergence of the Markov chains by inspecting the trace plots (see **SM**: Fig. S2b–S16b), Gelman–Rubin R and an estimate of the effective number of samples. In a Bayesian framework, each model conditions data on prior probability distributions and uses Monte-Carlo methods to generate posterior distributions for the parameters. The priors are the initial probabilities for each possible value of each parameter. We are able to visualize and interpret parameter estimates relative to a specific value by reporting and displaying the entire posterior distribution for each predictor and showing the highest density intervals (HDI’s) to reveal the most credible values for each parameter estimate. See **SM**: Fig. S1 for posterior distributions and HDI’s for the parameter religiosity for each model. Here we assume that 97.5% HDI’s which do not include zero are evidence that a parameter value is credibly different from the baseline.

### Model validity, effects, and specifications

To assess the validity of the models and their ability to reverse engineer the observed data, we conducted a posterior predictive check for each model (for results see **SM** Figs. S2a–S16a). Bayesian models are generative such that the posterior distributions (i.e., model predictions) produced by the models can be compared to the actual data. Hamiltonian Monte Carlo Chains programmed in STAN were used to generate posterior distributions. All models utilized broad but weakly regularizing priors that tamp down the effects of extreme values: normal distributions centered on 0 with a standard deviation of 1 for all fixed effects, a student-t prior with a mean of 3, a scale parameter of 2.3, and a shape of 2.5 to control the thickness of the tails. All models were run with four replicate chains for 1000 warm up and 5,000 MCMC iterations (See Github Models.R for all models and R code used).

## Results

**Figure 3:**
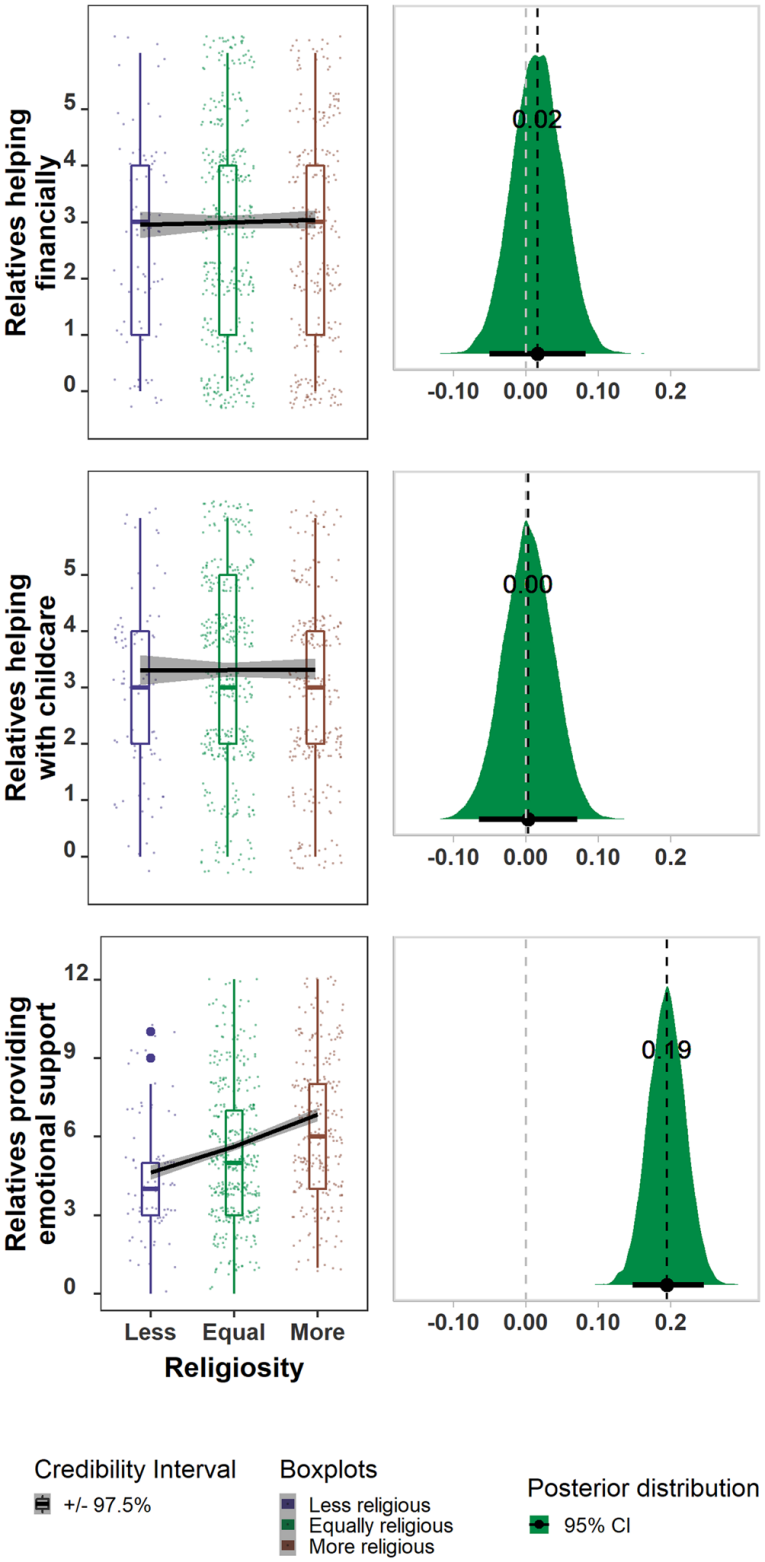
The number of relatives providing different types of help**.** More religious women do not receive additional financial support (top row) or childcare (middle row) from relatives but are more likely to receive emotional support from relatives (bottom row). Raw data points and error bars represent 97.5 percent credibility intervals for each category of increasing religiosity (left side) and the density interval for the respective posterior distributions and mean parameter estimates for religiosity for each model (right).

Women who report higher relative religiosity have larger social networks [supporting **P1a**] with more relatives in them [supporting **P1b**], both as a count and as a percentage, than those who report being less religious. Although the networks of more religious women are larger and more kin dense, they are also more geographically diffuse [failing to support **P2**]. Finally, more religious women report receiving more emotional support from their relatives [supporting **P3a**], but not more financial aid or help with childcare [failing to support **P3b** and **P3c]**. The parameter estimates and model results are shown in Table 1, Figs. 1, 2, 3 and **SM**: Fig. S1 (for full posterior distributions for religiosity).

### Network size and kin density

The total size of women’s social networks was positively associated with higher relative religiosity [**P1a**]. Our model predicts that women who report being less religious than other people in their village will have 8.9 people (97.5% CI 8.5–9.3) in their social network compared to 11.2 people (97.5% CI 10.9–11.6) for those with higher relative religiosity (Table 1, Fig. 1 (top panel); see also **SM**: Figs. S1, S2a and S2b).

The larger overall network size among more religious women was driven by a higher number and percentage of family members in these networks [**P1b**]**.** The number of non-relatives in women’s social networks, however, did not differ as a function of relative religiosity. Our model predicts an average of 1.03 unrelated people in a less religious woman’s social network (97.5% CI 0.86–1.23) compared to an average of 1.02 unrelated people (97.5% CI 0.91–1.17) for more religious women. In contrast, the number of relatives was predicted to increase by 2.5 individuals for more religious women, from 7.8 (97.5% CI 7.4–8.3) relatives in the networks of less religious women compared to 10.3 (97.5% CI 9.9–10.7) for the more religious. Finally, more religious women had a higher percentage of relatives in their social networks. Our model predicts that networks of less religious women will be composed of 82.3% (97.5% CI 79.8–84.9%) relatives (i.e., 18% non-relatives) while the networks of more religious women will be composed of 87.0% relatives (97.5% CI 85.1–88.9%) (Table 1, Fig. 1; see also **SM**: Figs. S1, S3a-S5a for posterior predictive check (PPC) and S3b-S5b for Markov chain (MC) plots).

### Geographic distance of kin and non-kin

Our findings show that women reporting higher religiosity have a larger number of relatives in their social networks living closer to them than less religious women, an average of 1.4 more relatives who are neighbors, 6.1 relatives (95% CI 5.8–6.4) for more religious women vs. 4.7 relatives (95% CI 4.4–4.9) for less religious women (**SM:** Fig. S17). A much less substantial pattern in the opposite direction is seen for non-relatives: less religious women have an average of 0.28 more non-relative neighbors in their social networks than more religious women—1.71 (95% CI 1.35–2.05) for the less religious vs. 1.42 (95% CI 1.28–1.66) for the more religious.

These results, however, are largely driven by the fact that more religious women have more relatives in their networks overall (see above*: Network size and kin density*)*.* In fact, relative religiosity was negatively associated with the geographic density of women’s social networks once we accounted for network size [**P2**]. This was true for both kin and non-kin networks. For example, our models show that the probability that less religious women will have relatives in their social network living in the same neighborhood is 82% (97.5% CI 77–89%) but this falls to 78% (97.5% CI 72–85%) for more religious women. A similar pattern is found for the probability that relatives will live in the same municipality, but a different neighborhood (5.5–11.0% for less religious women vs. 6.9–12.0% for more religious women), and for the probability that relatives will live in a different municipality (5.6–12.8% for less religious women vs 7.3–15.8% for more religious women). A similar, but somewhat stronger, pattern is seen for unrelated network members. Here the model predicts that less religious women will have a 92% (97.5% CI 87–96%) probability of non-kin in their social network living in the same neighborhood vs. 81% (97.5% CI 73–91%) for more religious women. Meanwhile, the probability that non-relative network members will live in the same municipality, but in a different neighborhood, is somewhat lower for less religious women (5.5–11% vs 6.9–12.0% for more religious women), as well as the probability that non-relative network members will live in a different municipality (5.6–12.8% for the less religious vs. 7.3–15.8% for the more religious). Together these results indicate that although more religious women have more relatives in their social networks living nearby, they also have more geographically dispersed networks with additional relatives living further away.

### Type of support received from relatives

More religious women have an average of 2.2 more relatives who provide them with emotional support than less religious women—6.8 (97.5% CI 6.5–7.0) relatives vs. 4.6 relatives (97.5% CI 4.4–4.9) [**P3a**]. Although it was not an a priori pre-registered prediction^61^, we also tested the difference in the number of relatives from whom respondents receive financial assistance and found no difference—3.0 (2.9–3.2) for the more religious vs. 3.1 (97.5% CI 2.9–3.3) for the less religious [**P3c**]. However, when we distinguish between large and small loans this pattern changes somewhat. Here more religious women receive slightly more large loans from relatives—0.95 (95% CI 0.81–1.09) for the more religious vs. 0.88 (95% CI 0.58–1.18) for the less religious but receive fewer small loans from relatives— 2.69 (95% CI 2.56–2.82) for the more religious vs. 2.46 (95% CI 2.19–2.73) for the less religious, though neither of these trends reached statistical significance. There was also no detectable difference in the number of relatives helping with childcare between more and less religious women [**P3b**]—3.2 (2.8–3.6) and 3.3 (2.8–3.8), respectively. Our model did, however, show *fewer* non-relatives helping with childcare for more religious respondents—0.38 (0.11–0.85) vs. 0.02 (0.00–0.04). See Table 1, Fig. 3 (all 3 panels) and **SM**: Figs. S1, S8a-S16a for PPC and S8b-S16b for MC plots and S18). Aside from the different patterns seen between large and small loans given and received, none of the other patterns changed substantially when we broke them down by their individual components (e.g., childcare help given vs childcare help received), likely because many types of support were reciprocal thus the given vs. received networks overlapped substantially. It should be mentioned, however, that even though higher religiosity was positively and significantly associated with all of the questions that we categorized as providing emotional support to relatives, the strongest relationship was for the number of relatives with whom they spent time.

### Interactions between religiosity and market integration

Although hypotheses relating to the interaction were not pre-registered, given the exceptionally rapid social and economic change that has occurred in Bangladesh in the past several decades, considering whether interactions between religiosity and market integration would affect our interpretation of results was important. We therefore decided to conduct a post-hoc analysis to determine whether an interaction between an individual’s religiosity and their level of market integration added explanatory power to any of our models by comparing WAIC scores to assess model weights. Models with interaction terms that received at least some model weight, as compared to models run without an interaction term, were interpreted as being useful to consider further (i.e., including the interaction terms in the model adds to our understanding of the relationship between religiosity and the dependent variable). Only five models run on the following dependent variables had interactions between our index measuring economic capital and religiosity that received any weight whatsoever in model comparisons: total size of network, number of relatives in network, geographic proximity to relatives, number of relatives providing emotional support, and the number of relatives helping with childcare. Of these, the only model in which the interaction term did not overlap with zero (and was therefore seen as interpretable) was the model predicting the number of relatives helping with childcare, which revealed a negative interaction between religiosity and economic capital (−0.08, 97.5% CI −0.16 to −0.02). In other words, less religious women who were wealthier (or conversely more religious women who were poorer) received more help from relatives with childcare. Overall, then, these results suggest little evidence of an important relationship between market integration and religiosity in this context.

## Discussion

These results suggest that, in certain contexts, religiosity may increase bonds among related women, with no concomitant decrease in relationships among non-kin. In rural Bangladesh**,** higher reported religiosity, as compared to one’s neighbors, is associated with larger, more kin-dense social networks and more emotional support from relatives. In contrast, we find no evidence that religion broadens social networks to include more non-kin or increases any type of support received from non-relatives. We also find that more religious women are more likely to have ties with individuals (both kin and non-kin) living outside of their neighborhood. In terms of our predictions, our main finding is that those who report being more religious have larger social networks [supporting **P1a**] with more relatives in them [supporting **P1b**]. In addition, more highly religious women have more relatives living near them. However, this is due to their larger size and they have more geographically diffuse networks overall [offering mixed support for **P2**]. Finally, more religious women report receiving more emotional support from relatives [supporting **P3a**] but do not report receiving any more help with childcare [failing to support **P3b**] or financial assistance [failing to support **P3c**]. Overall, these findings point to the important role that religion may play in fostering bonding social capital between relatives.

Together these results provide evidence that, in a rapidly developing economy with high levels of religious identification and practice, religiosity does not seem to broaden women’s social networks to include non-kin, but rather serves to reinforce and add to ties among relatives. As predicted, more religious women have larger social networks consisting of more relatives. These results are independent of family size in both the current and previous generation (number of siblings and number of children were entered as control variables in all models) and, unexpectedly, independent of multiple dimensions of market integration (three indices of market integration were entered as covariates). Furthermore, relatives seem primarily to provide emotional support while other kinds of help, such as childcare, are provided by neighbors regardless of kinship status. These results provide evidence that religiosity strengthens women’s relationships with kin while having no impact on the number or strength of relationships women have with non-relatives. In other words, among women, religiosity appears to build social capital by generating stronger bonding ties with family members rather than by broadening social networks to include more unrelated individuals or by replacing support networks of genetic kin with unrelated co-religionists^51^.

Therefore, if religion does promote cooperation among unrelated individuals^77,78^, it may not always do so by replacing kin relationships with relationships with unrelated co-religionists. Instead, our findings are consistent with research demonstrating the role that religion can play in solidifying relationships among family members^21,42,44^, indicating that religion may provide a buffer against fracturing kin networks in societies undergoing market integration^15,16^, at least during early phases of transition. These results further suggest that religion may help to strengthen bonds among kin who do not live near each other, and thus may play an important role in alleviating some of the other disruptive impacts that economic development can have on families impacted by labor migration and urbanization.

There was mixed support for the prediction that religious women will live nearer to relatives^61^. Although more religious women do seem to have more contact with relatives and, as a result, have a greater number of relatives from whom they receive support who live nearby, they also (in contrast to our original prediction) have more geographically dispersed social networks after adjusting for network size. In other words, although more religious women have more relatives living near them, this is largely the result of their having larger kin networks overall, as the overall spatial structure of their kin networks is more dispersed. Although this result was not predicted, it makes sense in the context of rapid economic development. In Bangladesh, which has undergone some of the most rapid market integration in the world in the past few decades^79^, geographic proximity may no longer be a particularly good indicator of the tightness of social networks^80^. This is because economic modernization provides new ways to interact through better communication and transportation systems^81^, and modern technology, like mobile phones, email, and social media^82^ has led to an increasing ability to maintain relationships. Indeed, many of the network partners women reported in our fieldwork—especially for social support, but also for loans—were connections operating most actively through mobile phones alongside occasional visits.

The wider geographic distribution of the social networks of more religious women might also help to explain why they receive emotional support from more relatives as geographic distance is expected to be less disruptive for emotional connections between kin than it is for non-kin^83^. A study of single mothers in urban Kenya, for example, showed that the road distance to their own mother had no effect on the amount of emotional support they received^84^, and other studies have suggested that it is less costly to maintain emotionally close relationships with relatives^85–87^. If religion increases social cohesion^53,54^ and strengthens social networks overall^56^, then it may also make it easier to maintain emotional connections with increasingly physically distant relatives. Our finding that more religious women do not receive help with either childcare or financial assistance from a larger number of relatives than less religious women may also be related to the greater geographic diffusion of their networks. Consistent with this possibility are results showing that more religious women receive more large loans and fewer small loans from relatives (a non-significant trend). In some ways, large loans and emotional support are similar in that they are not geographically determined. Both can be transferred across long distances (i.e., sending money via mobile banking or talking on the phone) between relatives who live very far apart, which is common in rural Bangladesh, as many women are married to men who spend significant portions of their time living in large cities (e.g., Dhaka, Chittagong) or abroad (e.g., Dubai, Saudi Arabia, Singapore). More religious women, who have connections with more relatives, may therefore be better able to tap into these types of support irrespective of geographic distance. There may also be a greater sense of obligation with large loans such that more religious women, who are closer with their families, may be more able to rely on relatives for these bigger financial commitments.

Although greater geographic diffusion might offer a partial explanation for these patterns, it is unlikely to explain all of them. This is because more religious women, owing to the fact that they have more kin in their networks overall, still have more relatives in their networks who are neighbors than less religious women (SM: Fig. S17). The fact that they still do not receive more help with childcare is consistent with the hypothesis that the childcare help they do receive does not depend on religiosity. Meanwhile, the counterintuitive finding that ***less*** religious women receive more childcare help from non-relatives suggests that less religious women may be enlisting more childcare help from unrelated neighbors—perhaps in response to the fact that they have fewer relatives close by or in their networks overall. An even simpler explanation for why more religious women do not seem to receive more help with childcare or financial assistance (i.e. small loans), however, may be that those who receive more support simply need more help^88,89^. Another analysis of the same women used in this study showed that those who received more help with childcare and housework often had worse nutritional status than those who gave such help^88^. This suggests that need is likely to be an important factor for receiving some types of help (e.g. financial and childcare help), while this pattern is less likely for other types of help such as emotional support^90^**.**

Although it was not the focus of this study’s predictions, the lack of any significant interaction effects between religiosity and level of market integration on social networks warrants some discussion. A common, but rarely directly tested^See 15 for exception^ assumption among researchers has been that as individuals move through the process of market integration, kin networks become weaker. This is expected, in part, because households entering the market economy often move from more intensive kinship patterns characterized by higher relatedness within family networks to more extensive kinship patterns characterized by lower relatedness within family networks^7^. Household autonomy also often increases in proportion to market-based opportunities to earn income^15^. In this case, more integrated households are often presumed to be better situated to tolerate fractured relationships with relatives^88,91^ which can result in a reduced reliance on kin. On the other hand, some have argued that if cooperative institutions are strongly kin-based^92^, or if relatives help people to access new market opportunities^93^, then this association may be reversed and there will be a positive relationship between the kin density of one’s social network and market integration*.* In contrast to either of these predictions, however, our findings suggest that a household's level of market integration changes neither how religiosity impacts the number of relatives in women’s social networks, nor the type or amount of support they receive. In other words, religiosity seems to be important for maintaining social relationships amongst relatives regardless of a household’s level of integration into the broader economy.

There are several reasons why this might be the case. Although there is good evidence that market integration is ultimately linked to declining religious beliefs, this association often occurs late in the demographic transition after fertility rates have already fallen below replacement levels and social safety nets have become common^59^. This is particularly true in Europe which has seen a rapid rise in the rate of non-believers since the 1960s^59^. In Bangladesh, however, recent, and substantial fertility declines^69^ have not been accompanied by significant reductions in religious practice. Although ethnographic observations and interview data suggest that more religious women are likely to pray more often, attend religious services and/or festivals more frequently, are more likely to adhere to religious standards of behavior, and generally feel that religion plays a more central or important role in their lives, nearly everyone still strongly identifies as either Muslim or Hindu and practices their religion^62^. In other words, there is little evidence of secularization in Bangladesh.

This has two important implications. First, countries like Bangladesh may not have as many secular organizations (e.g., social clubs, judiciaries, labor unions, and community associations)^94^ that some authors have argued helped to replace weakened religious institutions in western Europe and North America^60^. Therefore, the institutional and cultural changes that presaged and helped to smooth the transition from kin-based bonding social relationships to non-kin bridging relationships typical of late-stage market integration in Europe and North America have not taken place in Bangladesh. Thus, even if more market-integrated women are better able to withstand weakened ties with relatives, they may either have fewer options that would incline them to do so or may instead continue to derive significant benefit from cooperation with kin as key partners. This is consistent with our finding that most social network partners (83%) in this study were relatives. It is important to note, however, that it may become increasingly difficult to sustain this high level of kin density in future generations. Although the participants in this study have fewer siblings (and therefore fewer kin) than their parents, their children have even fewer siblings, and hence even fewer extended kin, as the fertility rate in Matlab fell rapidly from a Total Fertility Rate (TFR) over 6 in the 1970s to 2.7 by the early 2000s and has continued to fall, albeit at a more modest rate, in recent years^95^. While it is possible that future generations will have proportionally fewer kin in their networks (if they are able to maintain network size by replacing kin with non-kin), they may also experience an overall reduction in the size of their networks if these kin are not replaced. The relative lack of geographic mobility in Matlab (i.e., ~ 80% of social network partners live in the same neighborhood) may also play a role if most women are still living relatively close to relatives and therefore do not (yet) need to rely much on non-kin relationships. Indeed, the particular socio-cultural practice in rural Bangladesh by which patrilineally related extended families often live next to each other in small neighborhoods called *bari*s may play an important role in this process as well. These traditional living arrangements might help to slow the rapid transition from extended to nuclear families that has paralleled market integration in other parts of the world^96^ and suggest that local cultural practices are likely to have an important effect on how economic development affects social relationships^97^. Yet it is also true that this is a study of rural Bangladesh where the bari system is still a common feature of village life; baris are much less likely to persist in urban areas, suggesting we might see different structuring of social networks in urban Bangladesh.

Second, religious practices and traditions often promote family formation, and religious institutions both depend on and encourage relationships among relatives^59^. There is also evidence that more religious families get along better and like each other more than less religious families^21,22,39,40^. Because religious beliefs have not declined in rural Bangladesh, these pro-family cultural norms are nearly universally adopted and it is therefore not surprising that they do not vary with degree of market integration, particularly in rural Bangladesh. This echoes patterns in modern Iran and Saudi Arabia, both of which have undergone rapid and recent fertility transitions without much evidence of increasing secularism^59^. Although the overall culture is strongly family-oriented, more religious women may also hold more traditional social views, especially with respect to prescriptions regarding how to interact with kin or family members, which may help to explain these results. Indeed, the fact that almost all (94%) of the women surveyed self-identify as housewives and work inside the home (see SM text: *Data manipulation and categorization prior to Factor Analysis*) is likely to play an important role in maintaining relationships with kin. Although working outside the home may allow some Bangladeshi women income and autonomy, market employment has generally relied on families either having extensive help from kin or being wealthy enough to purchase help from a nanny and/or housekeeper to allow the family to manage the domestic and childcare tasks traditionally managed by wives. Women who work at home generally maintain close ties with their in-laws as well as their natal family in part by engaging in reciprocal networks of childcare and emotional support, while women who work outside of the home may face increased challenges maintaining these family ties or may in fact actively prioritize the creation of new ties instead^7^. In highly market-integrated Western societies, traditions of women working outside the home and relying on paid help rather than kin for childcare and domestic work may be one factor contributing to reduced kin density of social networks; it is possible that this transition may yet occur in the future in Bangladesh.

As expected, these results suggest that religiosity affects social relationships. More interesting, however, is the finding that among women in rural Bangladesh religiosity may help to strengthen or preserve emotional ties between relatives but does not serve to help expand social networks to include non-kin. The larger social networks of more religious women, full of more emotionally supportive kin, are likely to be an important advantage of religiosity, providing a range of potential benefits for the physical and mental health of these women^98–99^. Numerous studies have shown a positive relationship between religiosity and life satisfaction^100–102^ and emotional support^103^ often mediates this relationship^1^. Moreover, such networks can have important effects on basic demographic processes such as fertility and might even help to explain why more religious people have more children, as they have access to more instrumental support (allocare)^49^. In addition, in some countries emotional support may be a better predictor of fertility than either childcare or financial assistance^104^.

Although this research sheds light on the role of religiosity in maintaining relationships among kin, these data are not without limitations. One methodological choice which simplified our analysis and interpretation was to place all relatives (e.g., maternal, paternal, siblings, first cousins, in-laws etc.) in the same category. It is possible, indeed likely, that different types of relatives help in different ways. Recent research, for example, has suggested that market integration disrupts childcare from paternal, but not maternal kin who may be more likely to travel greater distances to help^105^. Although the age and specific relationships are available in these data and will be included in future research, the focus of this study was on broadly distinguishing between relatives and non-relatives rather than looking at more detailed ego network relationships. Finally, it is also important to remember that we only analyzed the social networks of women in a society that is highly segregated by sex and where many women have limited involvement in the market or public life. It is therefore likely that the patterns we report here may differ for men, and preliminary analyses of the social networks of the husbands of these women suggests that this is in fact the case (Lynch et al. in preparation).

## Conclusion

Overall, these results suggest that, in a rapidly developing economy, higher religiosity among women serves to strengthen relationships between relatives rather than to expand social networks to include more unrelated co-religionists^51^. As compared to less religious women, more religious women have larger social networks, more relationships with kin, more geographically widespread networks and receive more support that is not spatially dependent, specifically emotional support and large loans. These findings shed light on the important role religiosity can play in building bonding social capital and how religion may buffer some of the effects of increasing geographic dispersal of kin networks that often occurs during market integration. Faith communities are extraordinarily potent repositories of social capital^33^, and secularization has been cited as one of the leading causes of declining social capital in Western democracies^106^. Religious beliefs, practices and institutions may therefore help to moderate the effects of changing social networks. These results point to religion’s function in maintaining relationships amongst families as nations navigate the dynamic and potentially destabilizing process of globalization. The role of religion in building and maintaining bonding relationships between relatives also suggests a potential alternate course for the trajectory of social networks in areas currently undergoing economic development. In places where social networks are still largely kin-based, and there is little evidence of secularization (i.e., religion maintains a central role in cultural norms, behavior and institutions), more religious women may not need to rely on relationships with non-kin co-religionists^23,52^ when they can count on ongoing relationships with kin.

## Supplementary Information

Supplementary Information.

## Data Availability

The datasets used and analysed in the current study are available in a Github repository.
