# Supplementary material for: Religiosity is associated with greater size, kin density, and geographic dispersal of women’s social networks in Bangladesh
Source: Sci Rep. 2022 Nov 5;12:18780. doi: 10.1038/s41598-022-22972-w (PMC9637216; doi:10.1038/s41598-022-22972-w)
Supplement: Supplementary file 1 — Supplementary Information. [file 41598_2022_22972_MOESM1_ESM.docx]

**Supplementary Materials:**

**Religiosity is associated with the size, kin density, and geographic dispersal of women’s social networks in Bangladesh**

**MARKET INTEGRATION**

Globalization is fundamentally altering social relationships as modernization increases geographic mobility. This process parallels the pace of the demographic transition to smaller family sizes [(Zelinsky, 1971)](https://paperpile.com/c/duVn8u/tBuv6) and as more people migrate further from their place of birth seeking work or education, they are more likely to leave the reduced number of relatives they have left behind [(Wilding, 2018)](https://paperpile.com/c/duVn8u/WilYo). Together lower fertility and greater geographic mobility are frequently associated with a transition away from extended families and towards nuclear families, a transition to wage-labour, increasing access to education, lower mortality rates and increasing investment in human capital [(R. A. Godoy et al., 2004; Gurven et al., 2015; Kaplan, 1996; Zelinsky, 1971)](https://paperpile.com/c/duVn8u/tBuv6+CFPEr+3bDiL+3OOi0). In communities transitioning from subsistence farming to market-dependence in rural Poland, for example, degree of market integration was associated with less kin-dense social networks [(Colleran, 2020)](https://paperpile.com/c/duVn8u/GSOef). It is important to note, however, that because modern communications systems allow people to stay in contact with relatives who live far away, social networks may not be as dependent on geographic proximity as in previous eras and in places that are more connected to the global marketplace and modern technologies [(Viry, 2012)](https://paperpile.com/c/duVn8u/iXwsP). Indeed, some evidence suggests that geographic distance is not as disruptive to relationships between close relatives as it is for more distant relatives or nonkin [(Pollet et al., 2013)](https://paperpile.com/c/duVn8u/Ju62R). Nevertheless, these changing relationships and networks can have important effects on behavior, including fertility decisions, and more interactions with non-kin have been shown to increase the spread of values associated with lower reproduction [(Newson et al., 2007)](https://paperpile.com/c/duVn8u/eBTFJ). The integration of traditional societies into market economies is characterized by the commodification of labor, capital, land, and goods and services which can be measured in a variety of ways (Lu 2007).

**References**

1. [Kolenikov, S. & Angeles, G. SOCIOECONOMIC STATUS MEASUREMENT WITH DISCRETE PROXY VARIABLES: IS PRINCIPAL COMPONENT ANALYSIS A RELIABLE ANSWER? *Rev. Income Wealth* **55**, 128–165 (2009).](http://paperpile.com/b/vV9ZiB/ubZh)

2. [Costello, A. B. & Osborne, J. Best practices in exploratory factor analysis: Four recommendations for getting the most from your analysis. *Practical assessment, research, and evaluation* **10**, 7 (2005).](http://paperpile.com/b/vV9ZiB/JT2m)

3. [Braveman, P. A. *et al.* Socioeconomic status in health research: one size does not fit all. *JAMA* **294**, 2879–2888 (2005).](http://paperpile.com/b/vV9ZiB/lGrk)

4. [Colleran, H., Jasienska, G., Nenko, I., Galbarczyk, A. & Mace, R. Fertility decline and the changing dynamics of wealth, status and inequality. *Proc. Biol. Sci.* **282**, 20150287 (2015).](http://paperpile.com/b/vV9ZiB/HACb)

**Tables and Figures:**

**
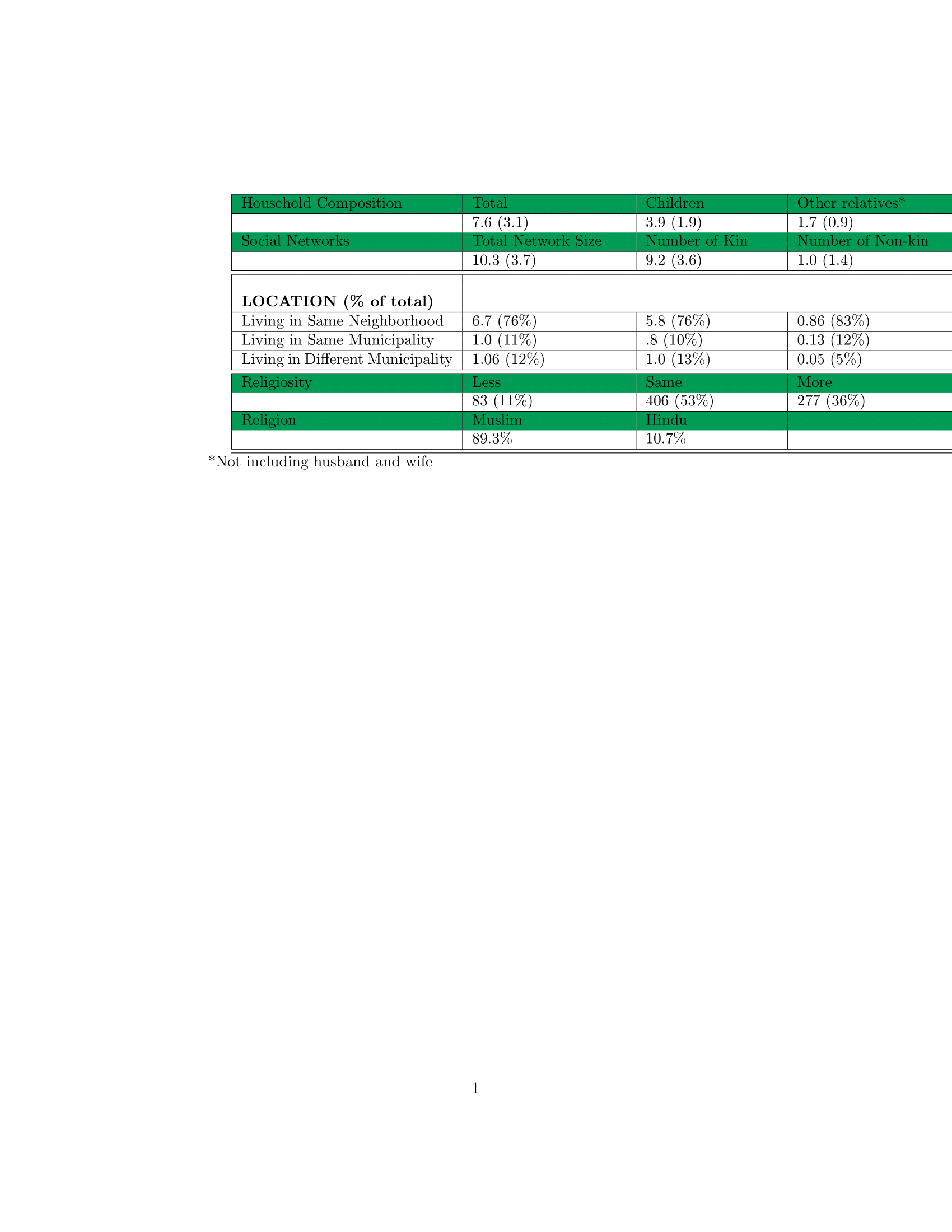
**

**Table S1: Descriptive statistics for Household and social network composition and Religion,**


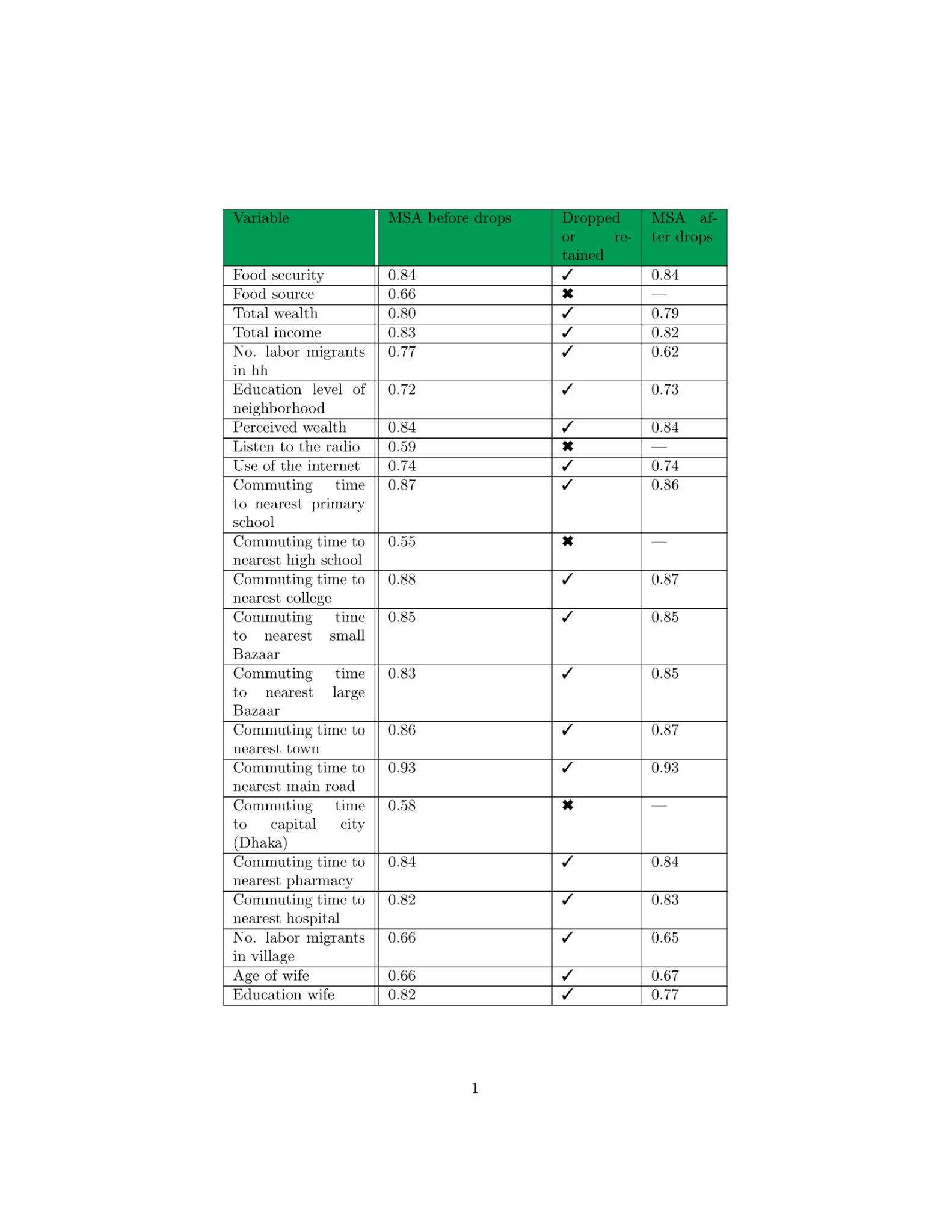

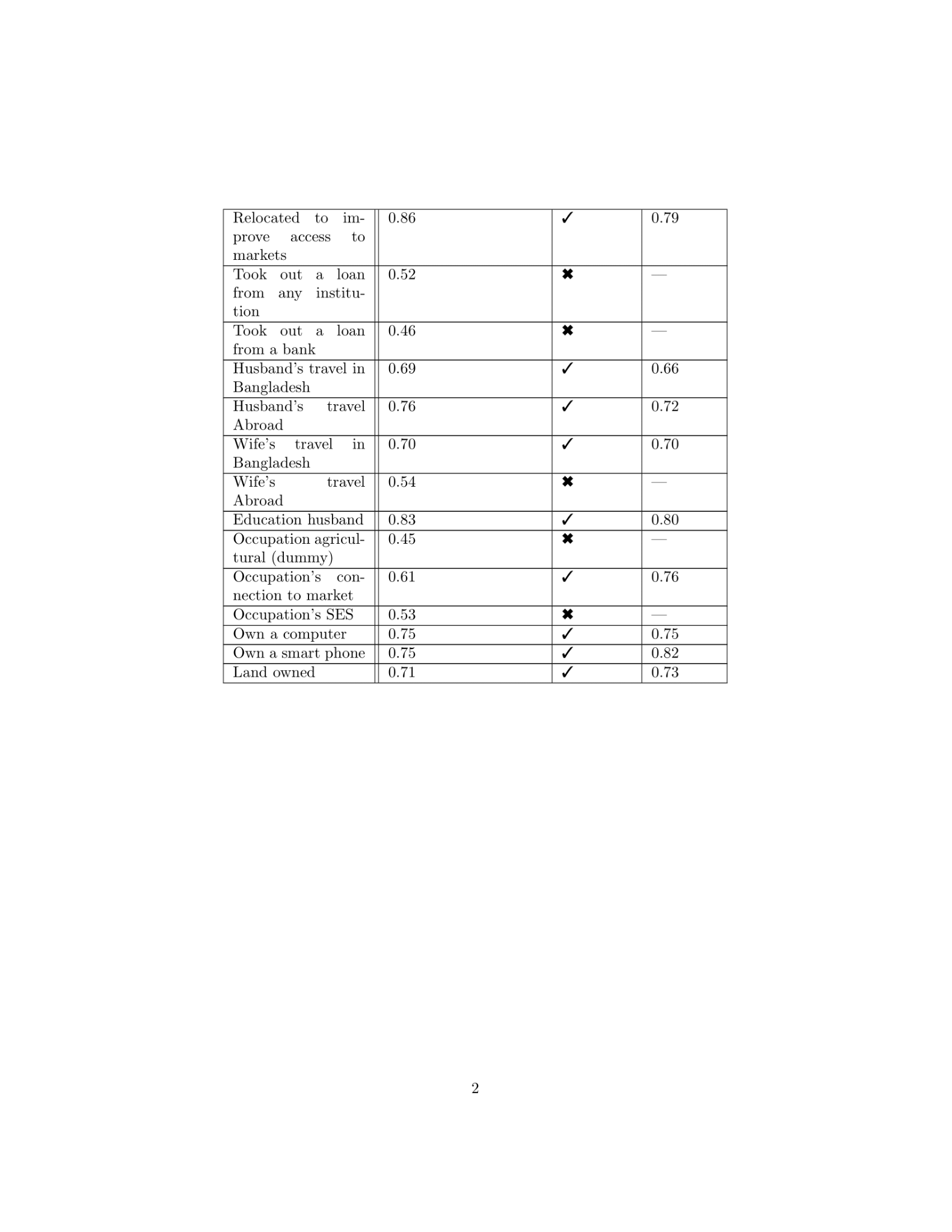


**Table S3:** Kaiser-Meyer-Olkin test: measures for sampling adequacy (MSA) before and after dropping data.

**
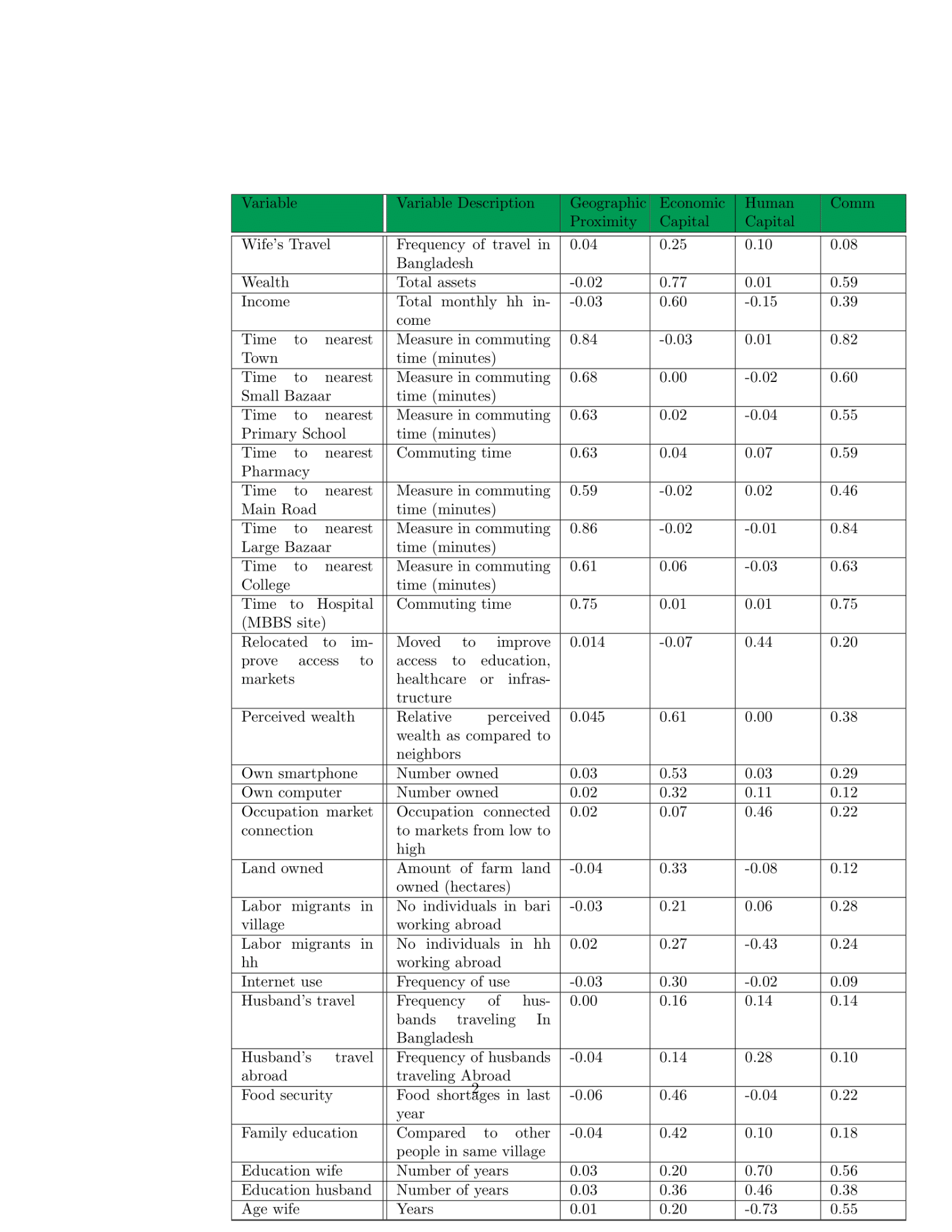
**

**Table S4:** Variables, their descriptions, factor loadings for each of the 3 extracted factors( M1: Geographic proximity to markets (commuting time) M2: Economic capital (wealth and assets) and M3: Human capital (education and occupation) and commonalities (how much variance in the measured variable is captured by the extracted factors).

**
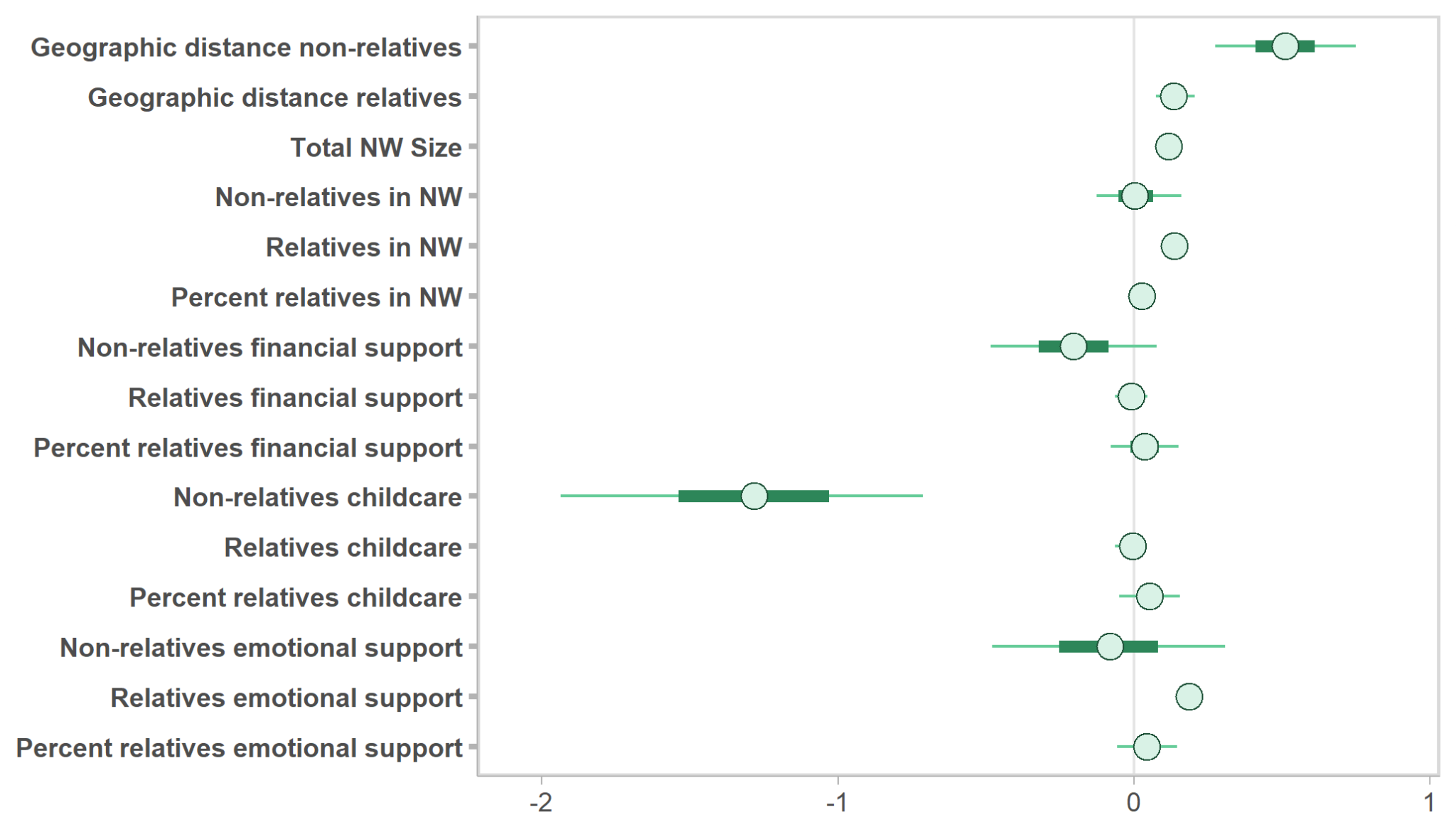
**

**Figure S1 Posterior distributions:** Religiosity score on X axis for each model and DV (Y -axis)


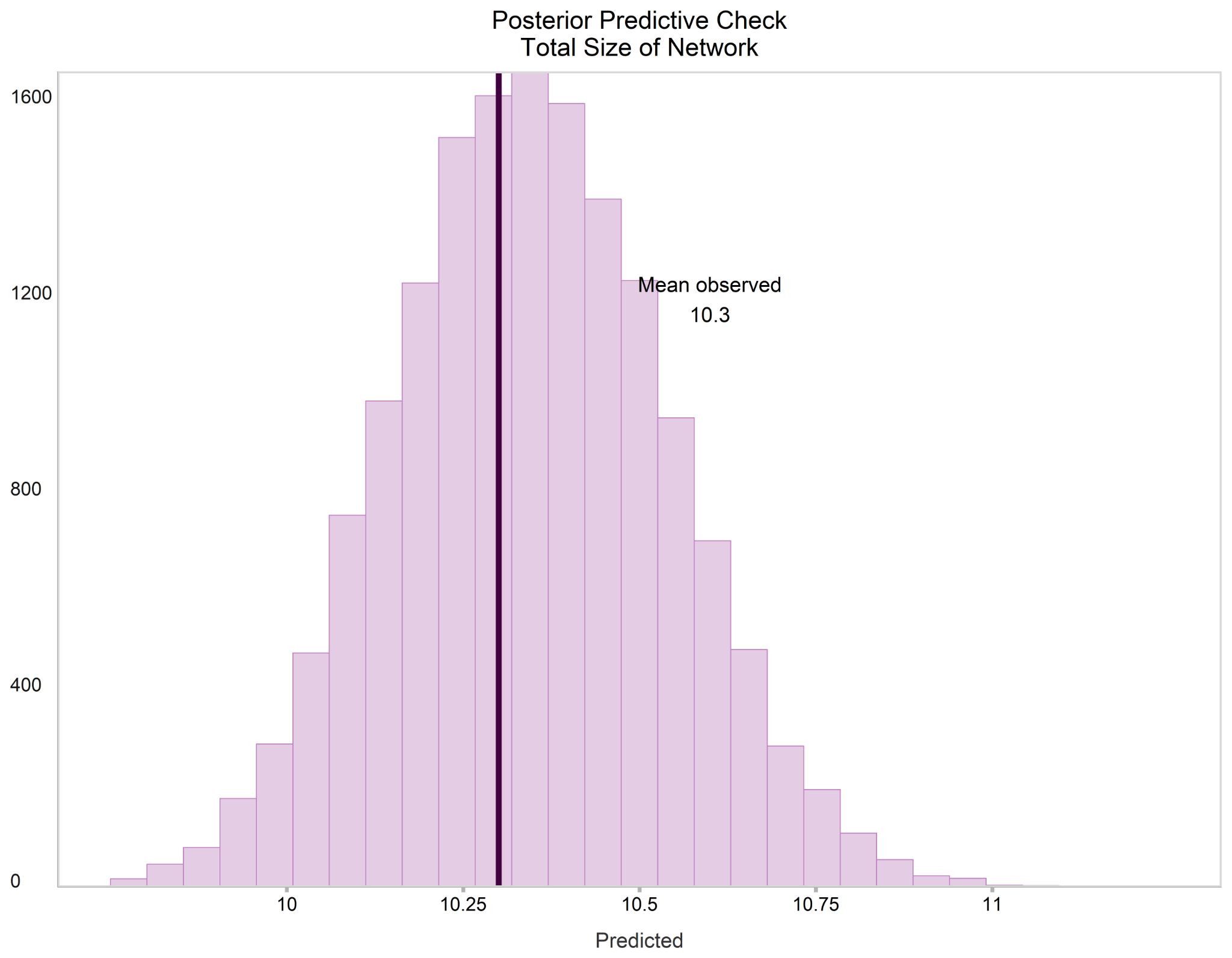


**Figure S2a** Posterior predictive check, Total size of NW


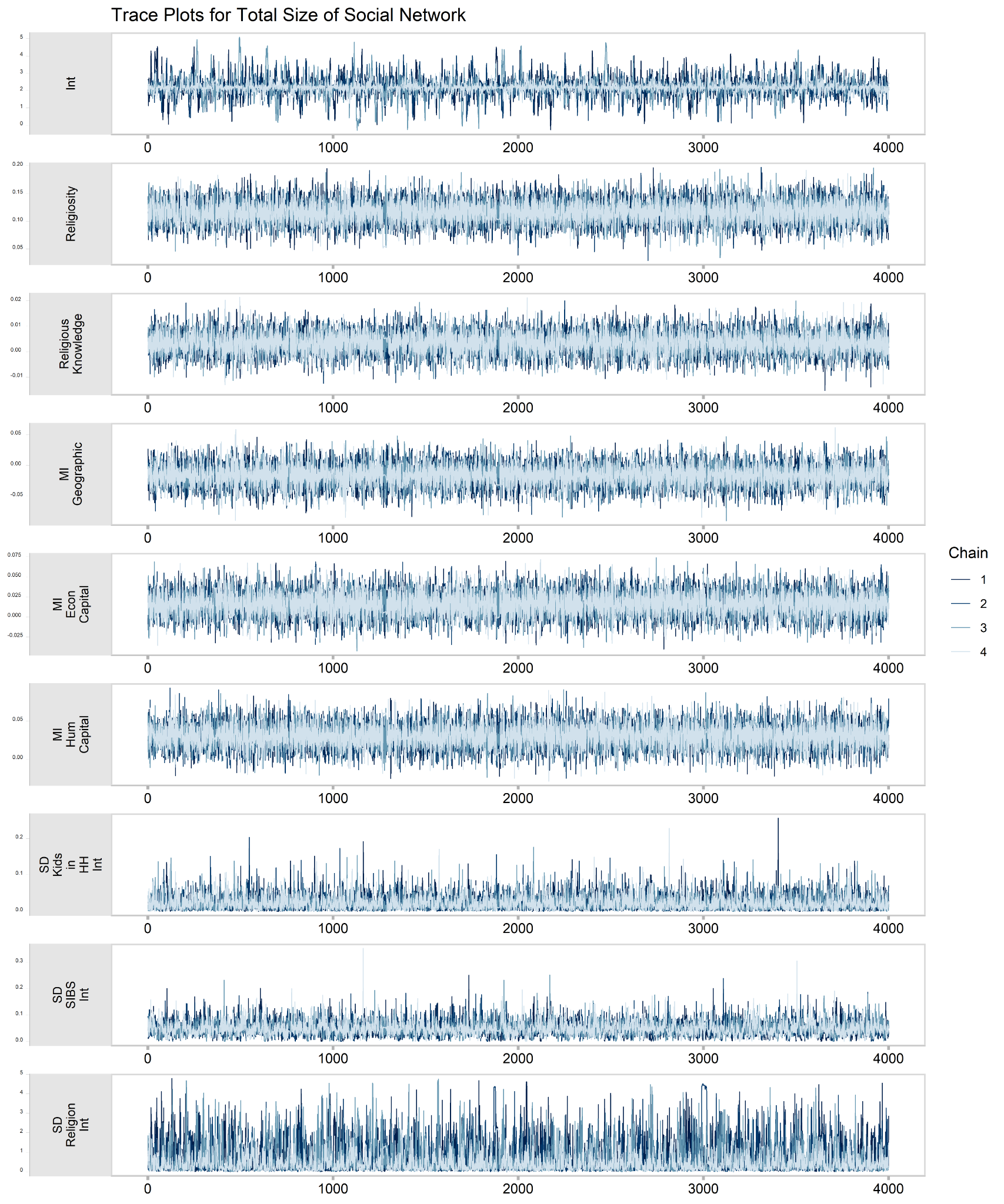


**Figure S2b**  Markov Chains, Total size of NW


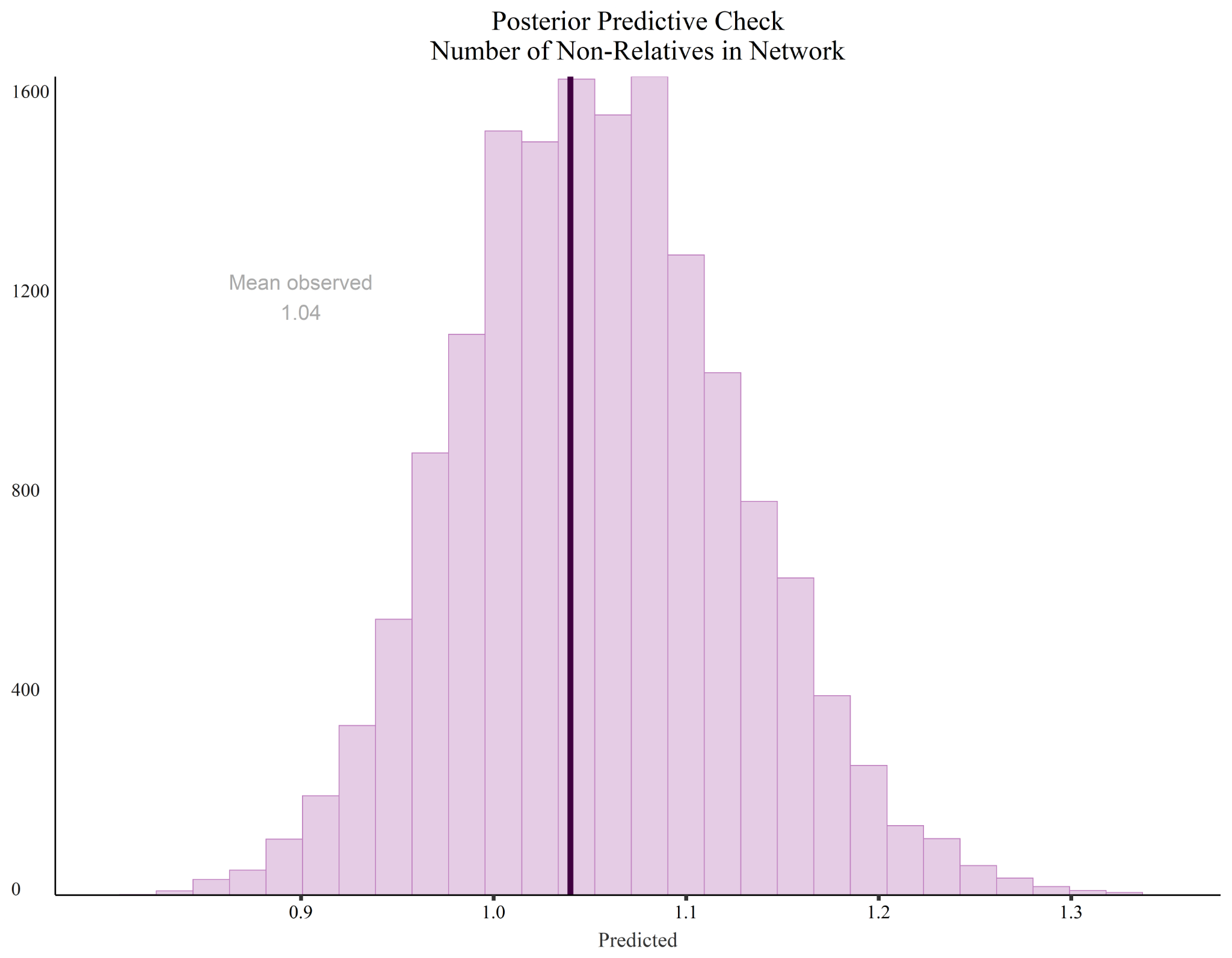


**Figure S3a** Posterior predictive check, Number of non-relatives in NW


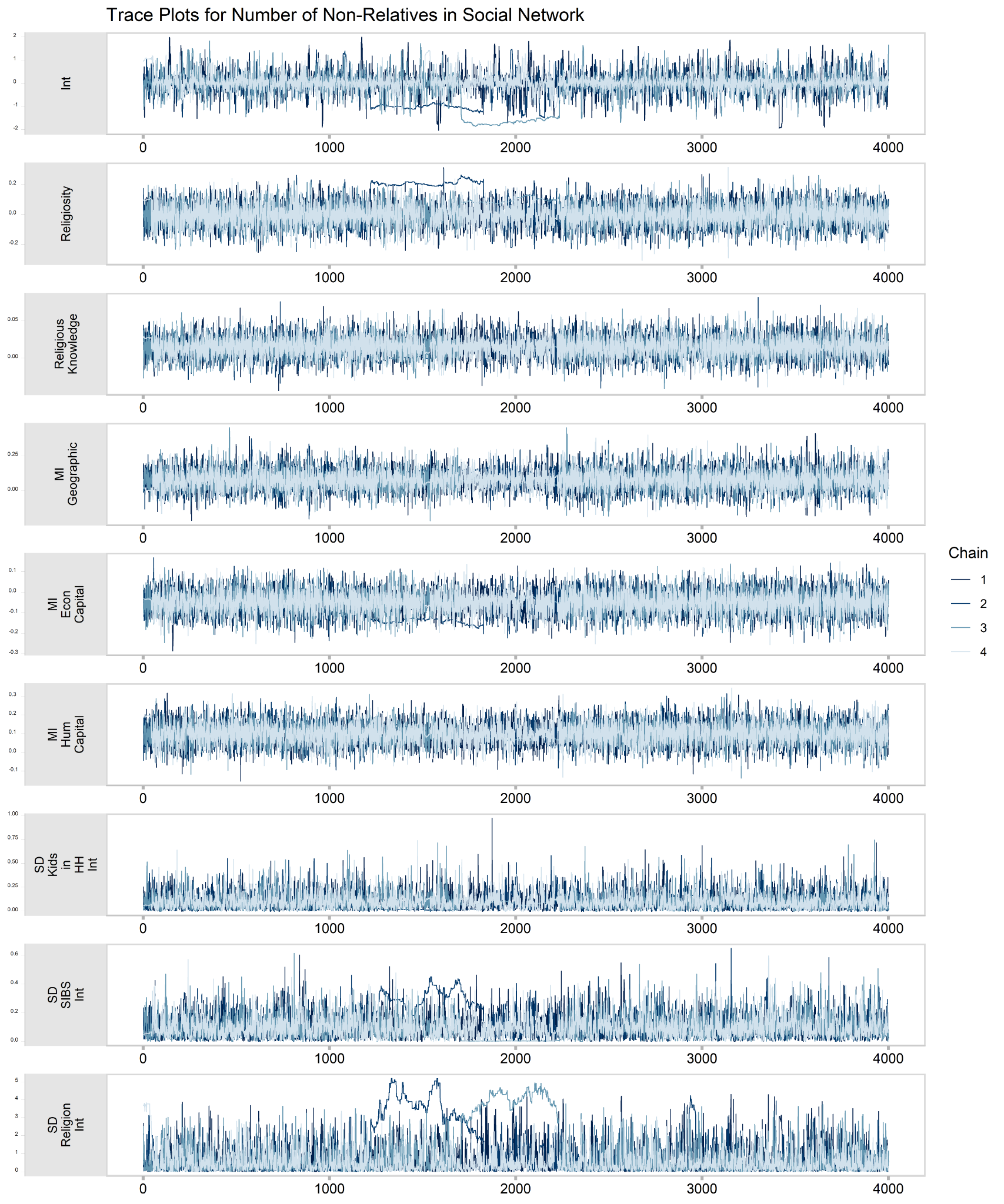


**Figure S3b** Markov Chains, Number of non-relatives in NW


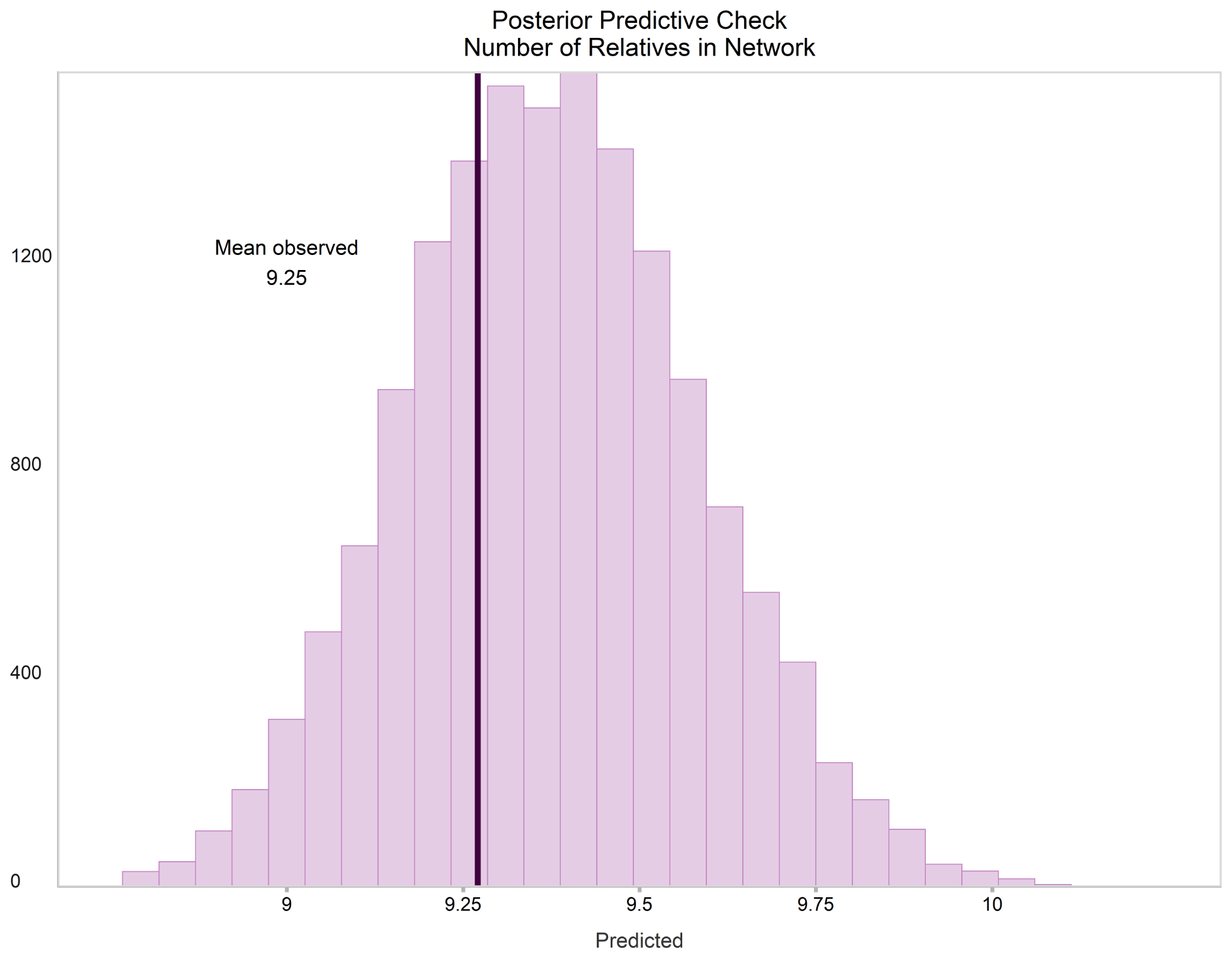


**Figure S4a** Posterior predictive check, Number of relatives in NW


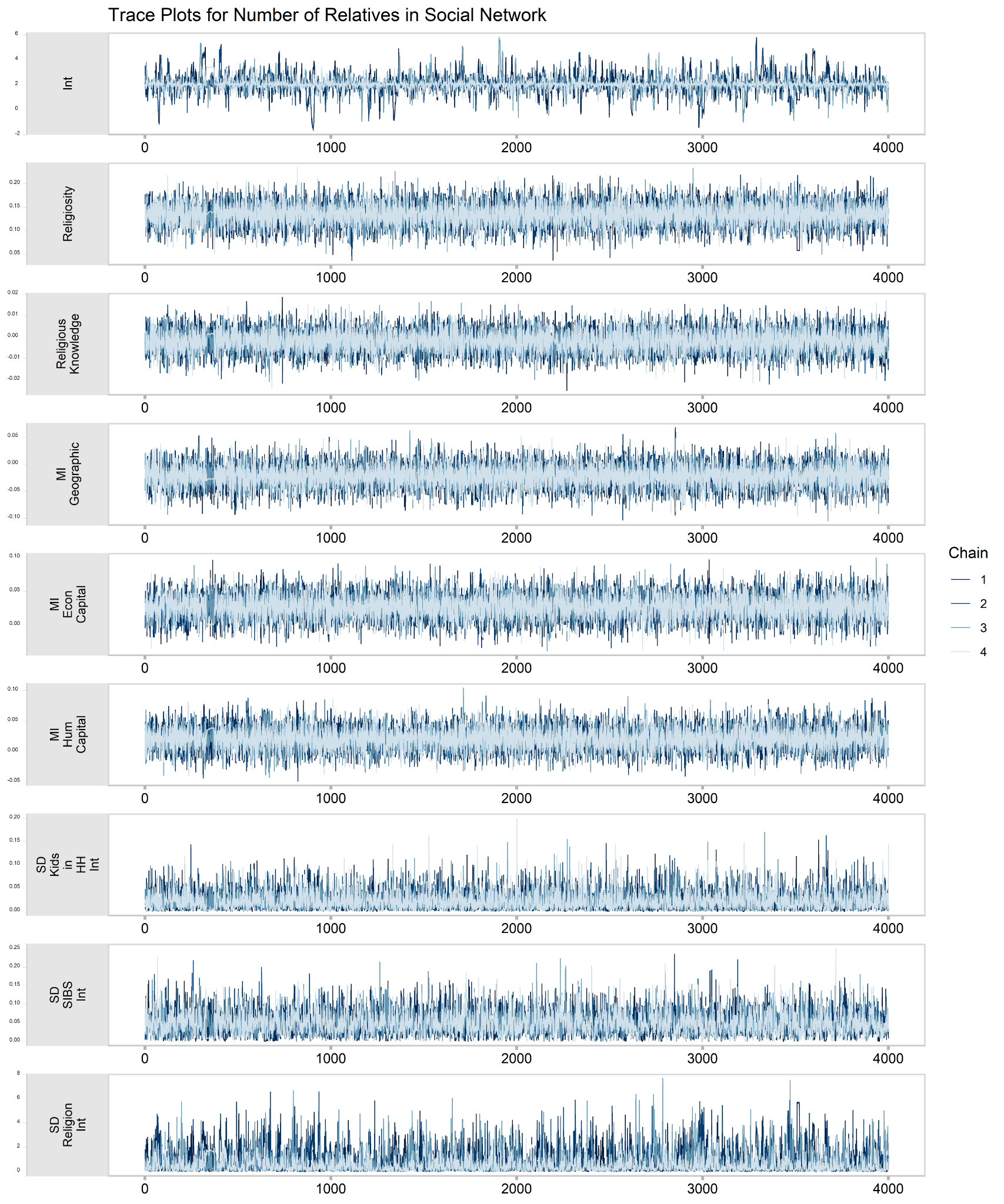


**Figure S4b** Markov Chains, Number of relatives in NW


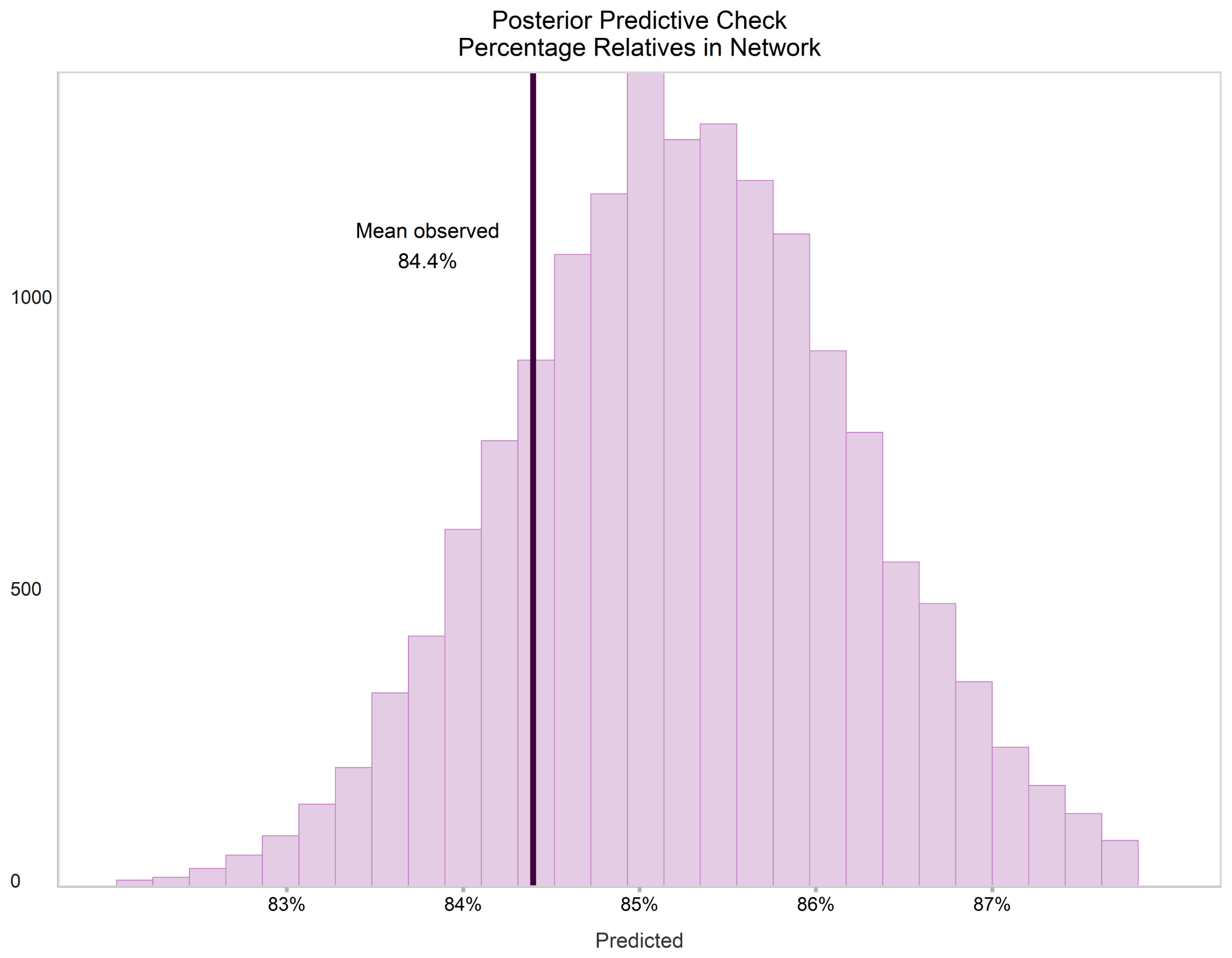


**Figure S5a** Posterior predictive check, Percentage of relatives in NW (PPC6)


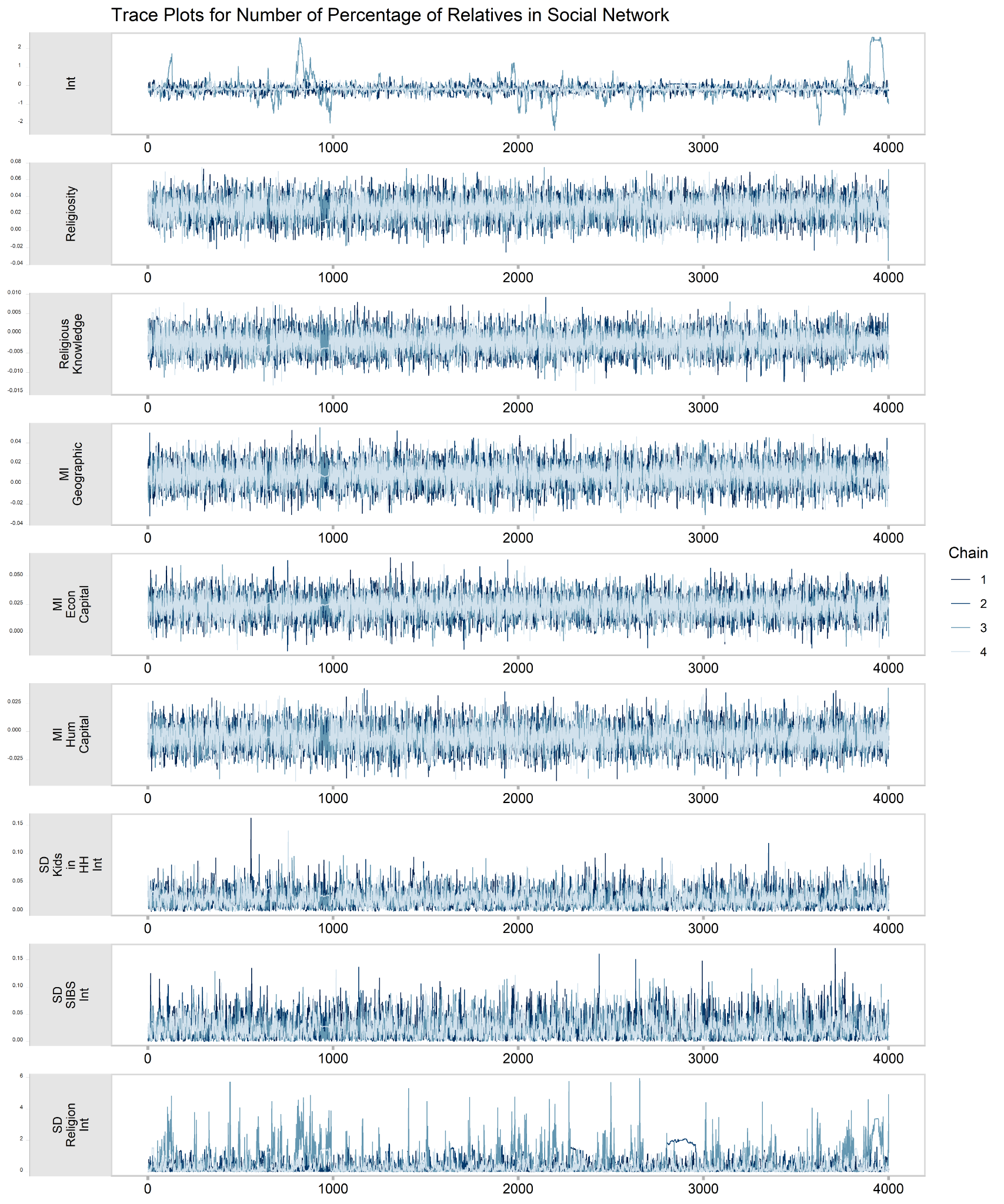


**Figure S5b** Markov Chains, Percentage of relatives in NW (Trace_plot_6)


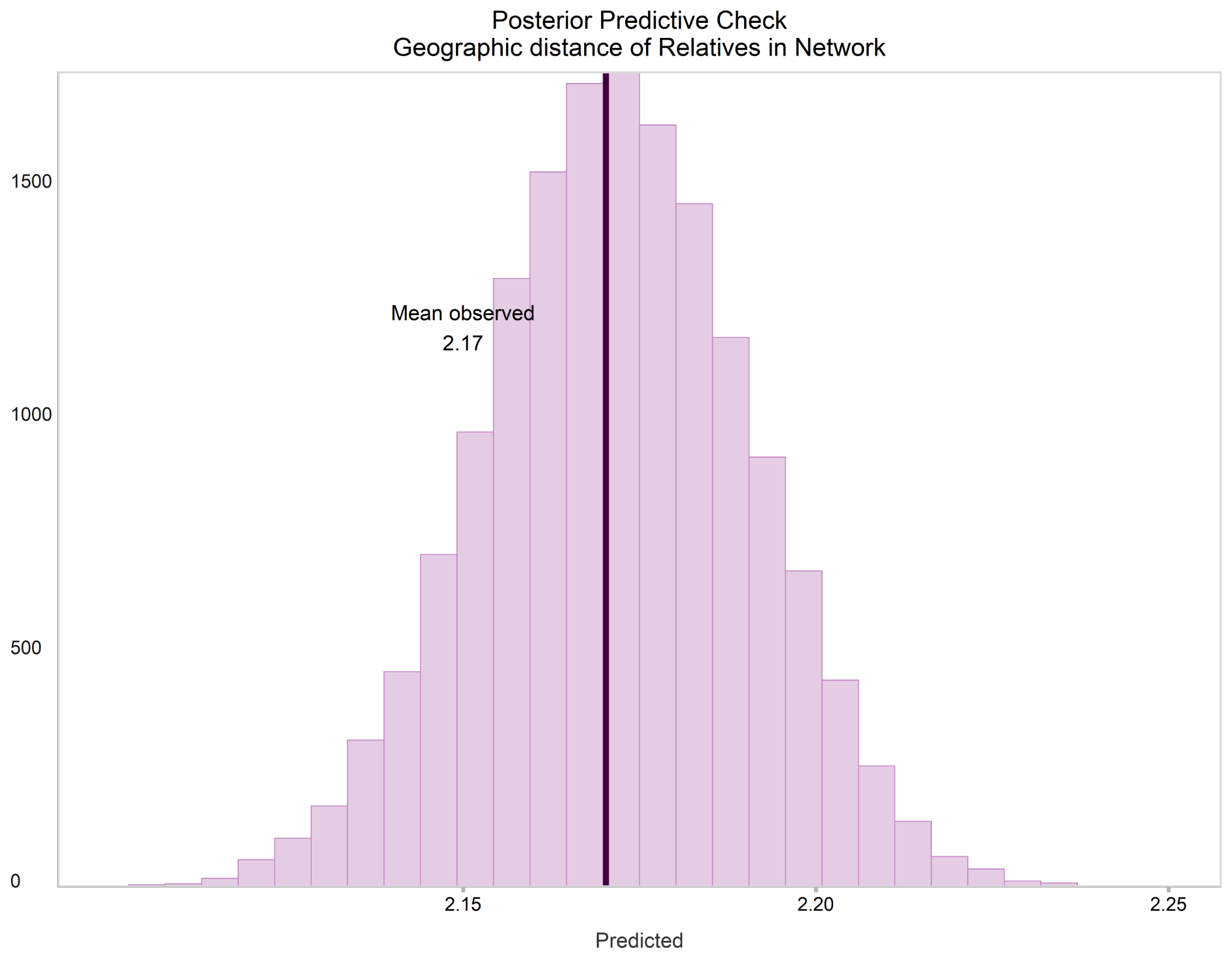


**Figure S6a** Posterior predictive check, Geographic distance of relatives (PPC2)


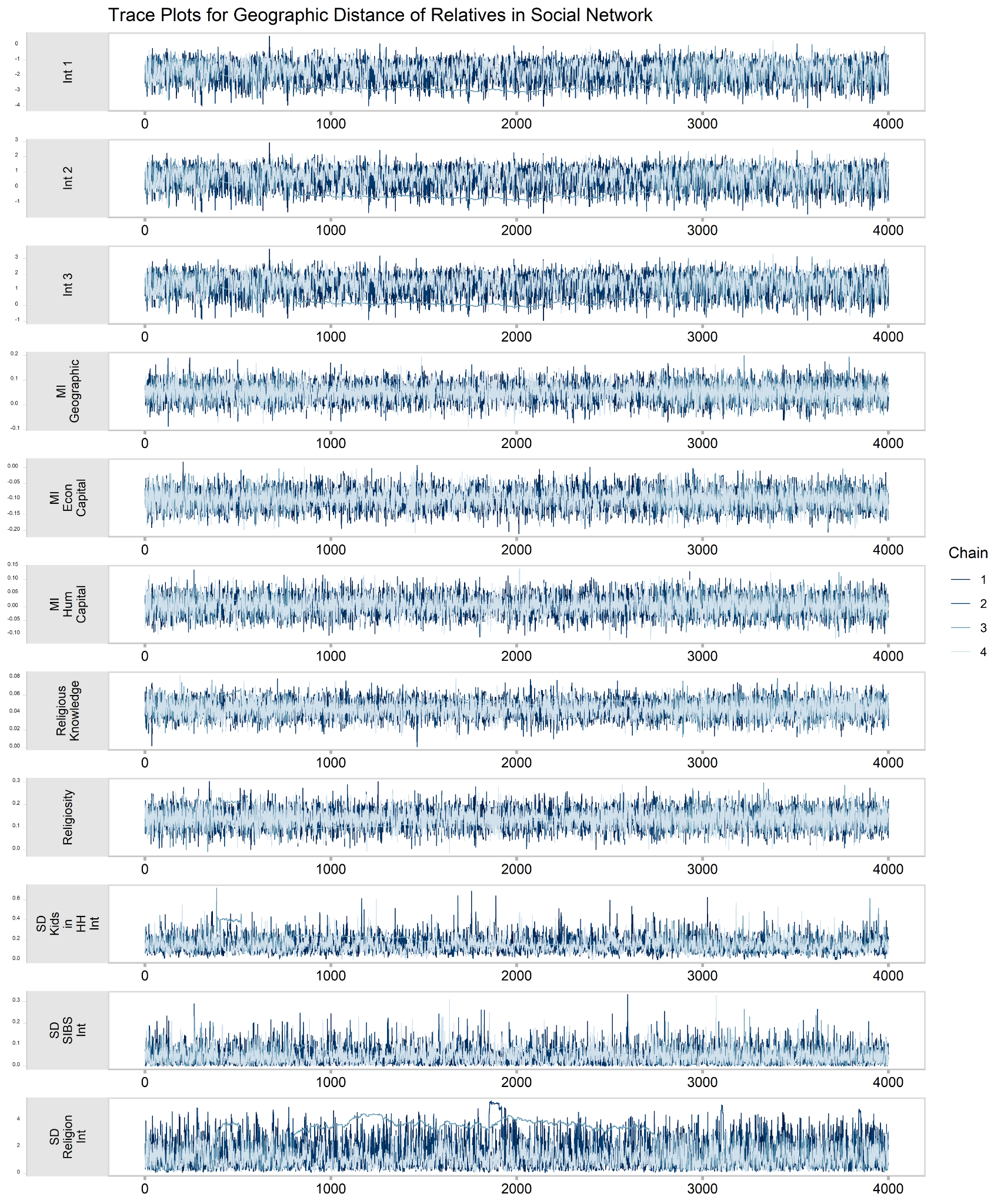


**Figure S6b**  Markov Chains, Geographic distance of relatives (Trace_plot_2)


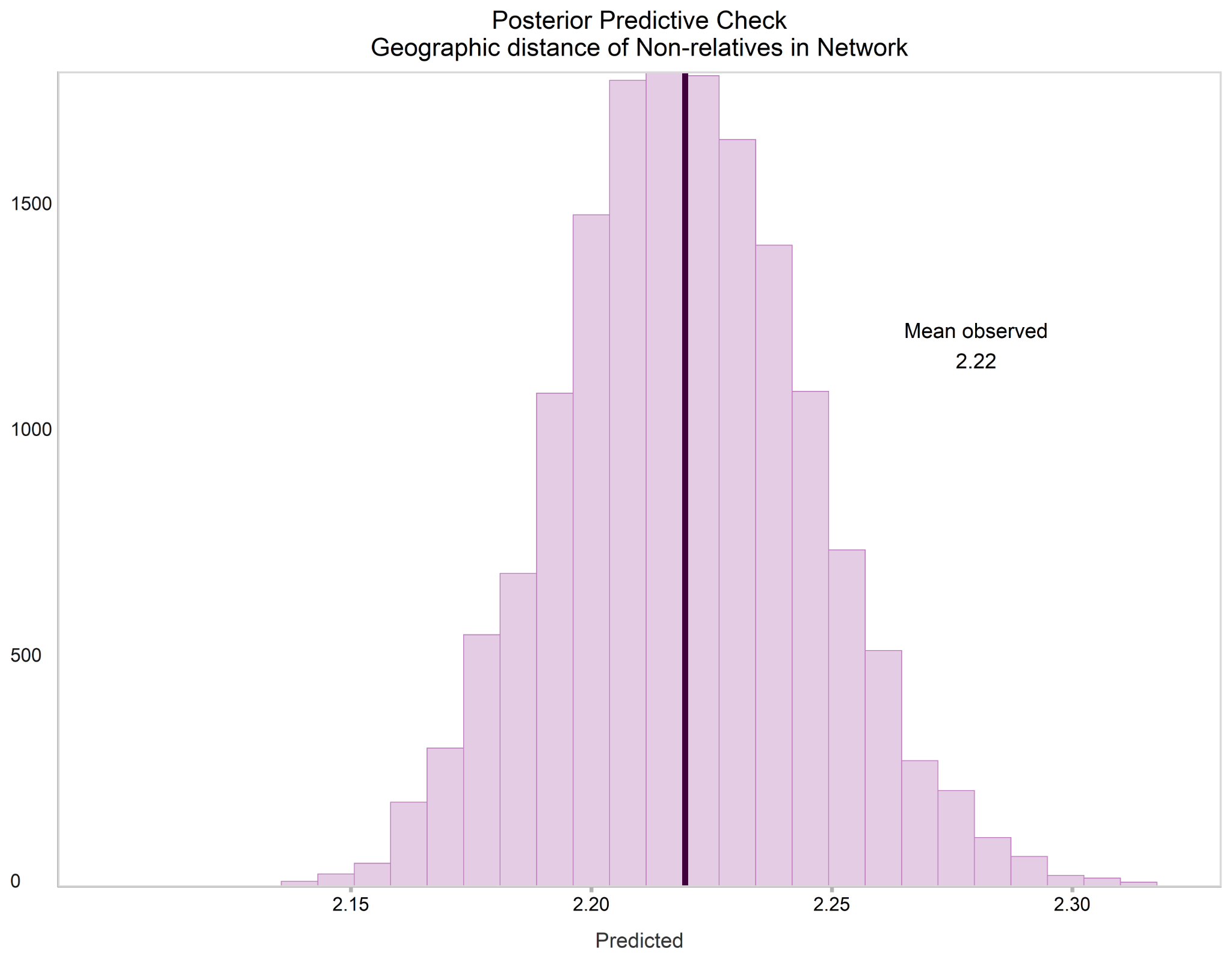


**Figure S7a** Posterior predictive check, Geographic distance of non-relatives (PPC1)


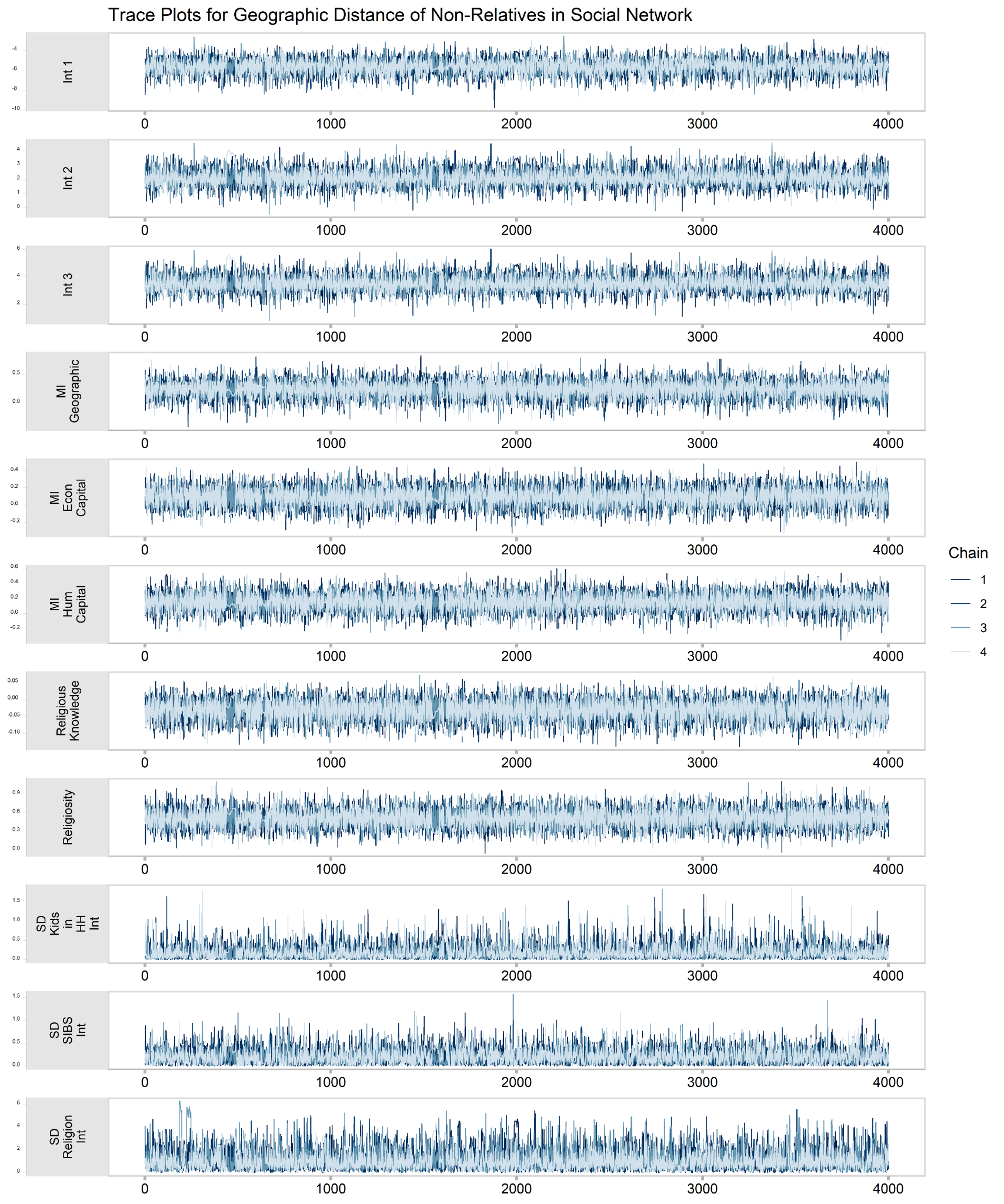


**Figure S7b** Markov Chains, Geographic distance of non-relatives. (Trace_plot_1). Variables entered as random effects not shown.


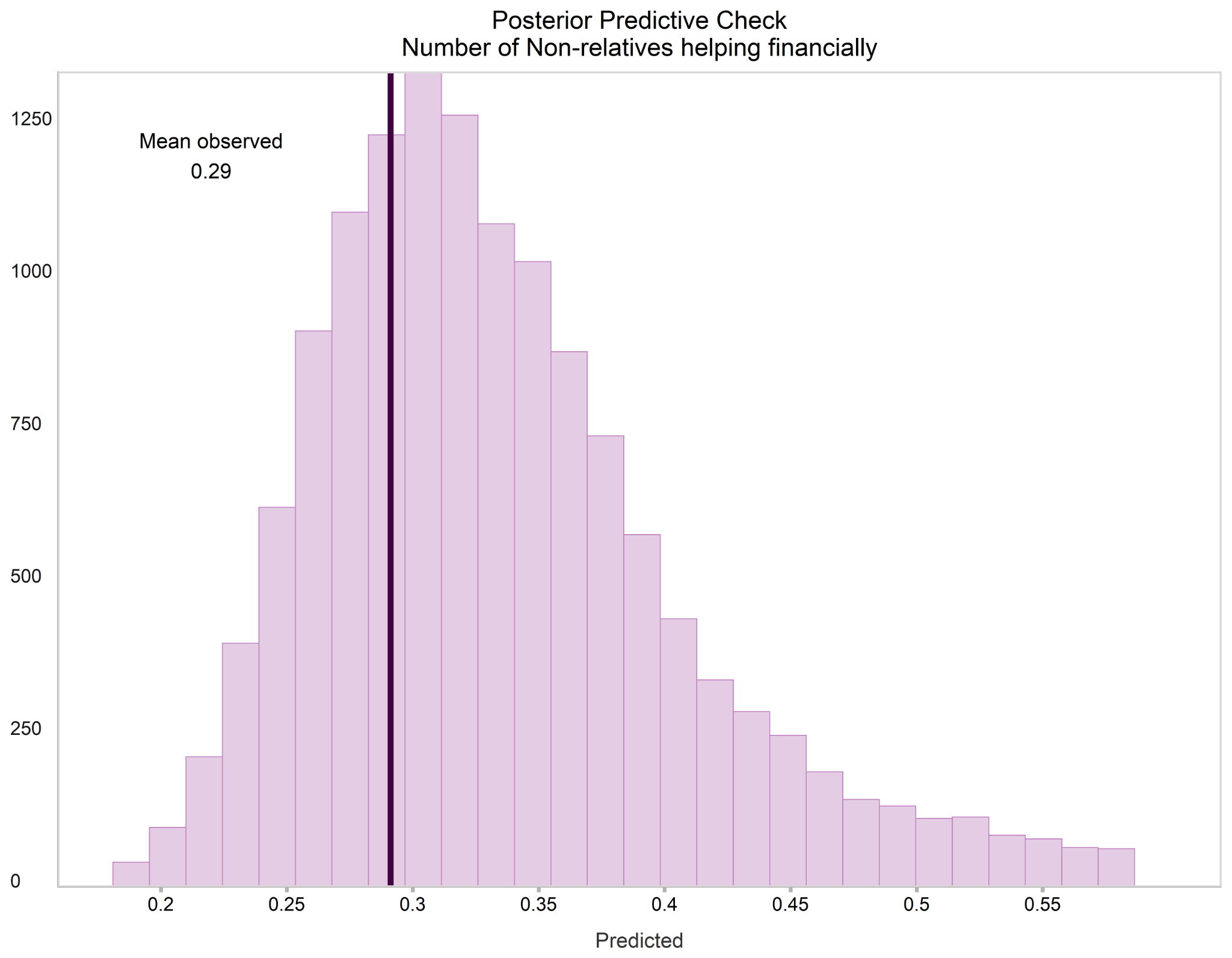


**Figure S8a** Posterior predictive check, Number of non-relatives providing financial support (PPC7)


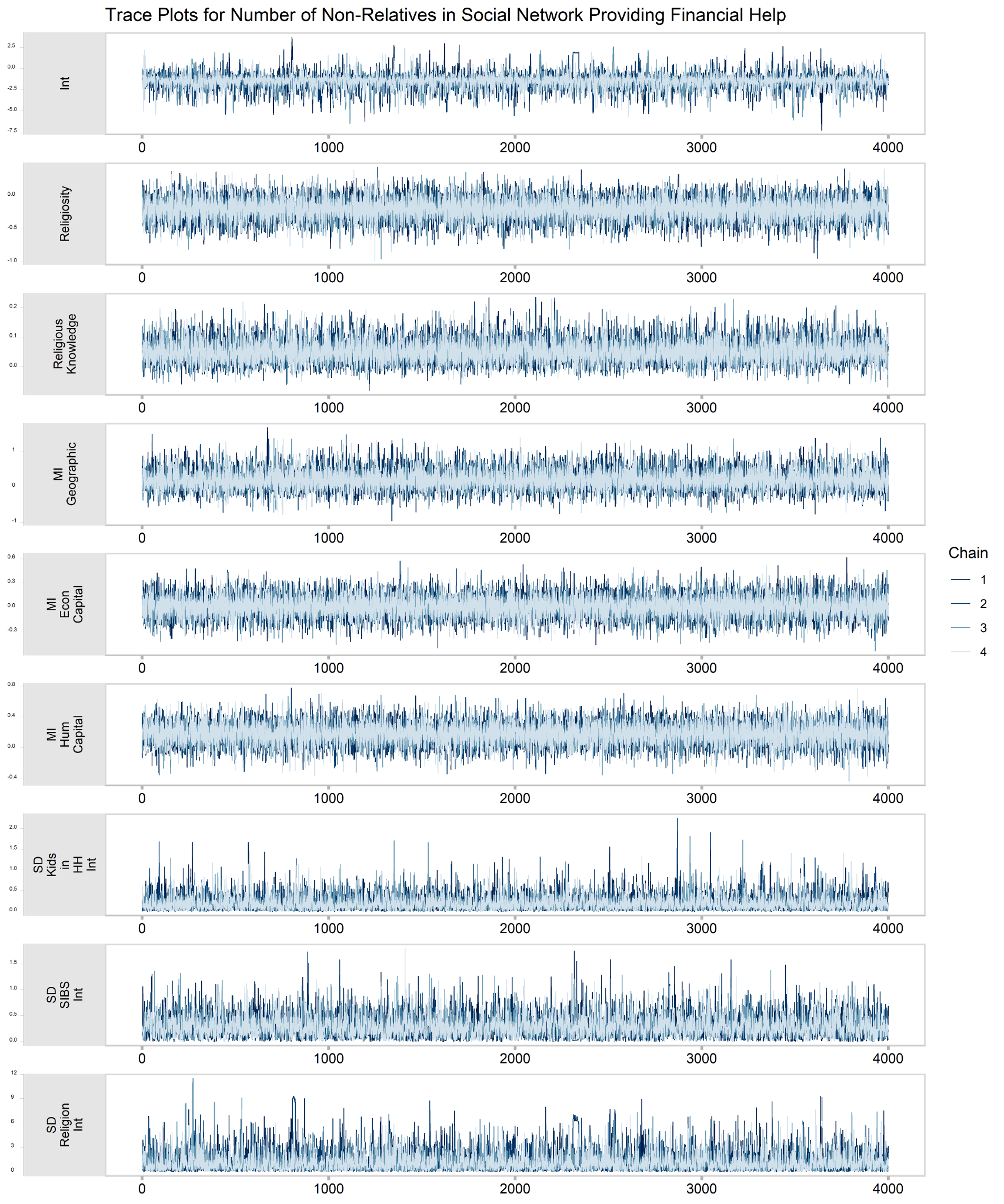


**Figure S8b** Markov Chains, Number of non-relatives providing financial support (Trace_plot_7)


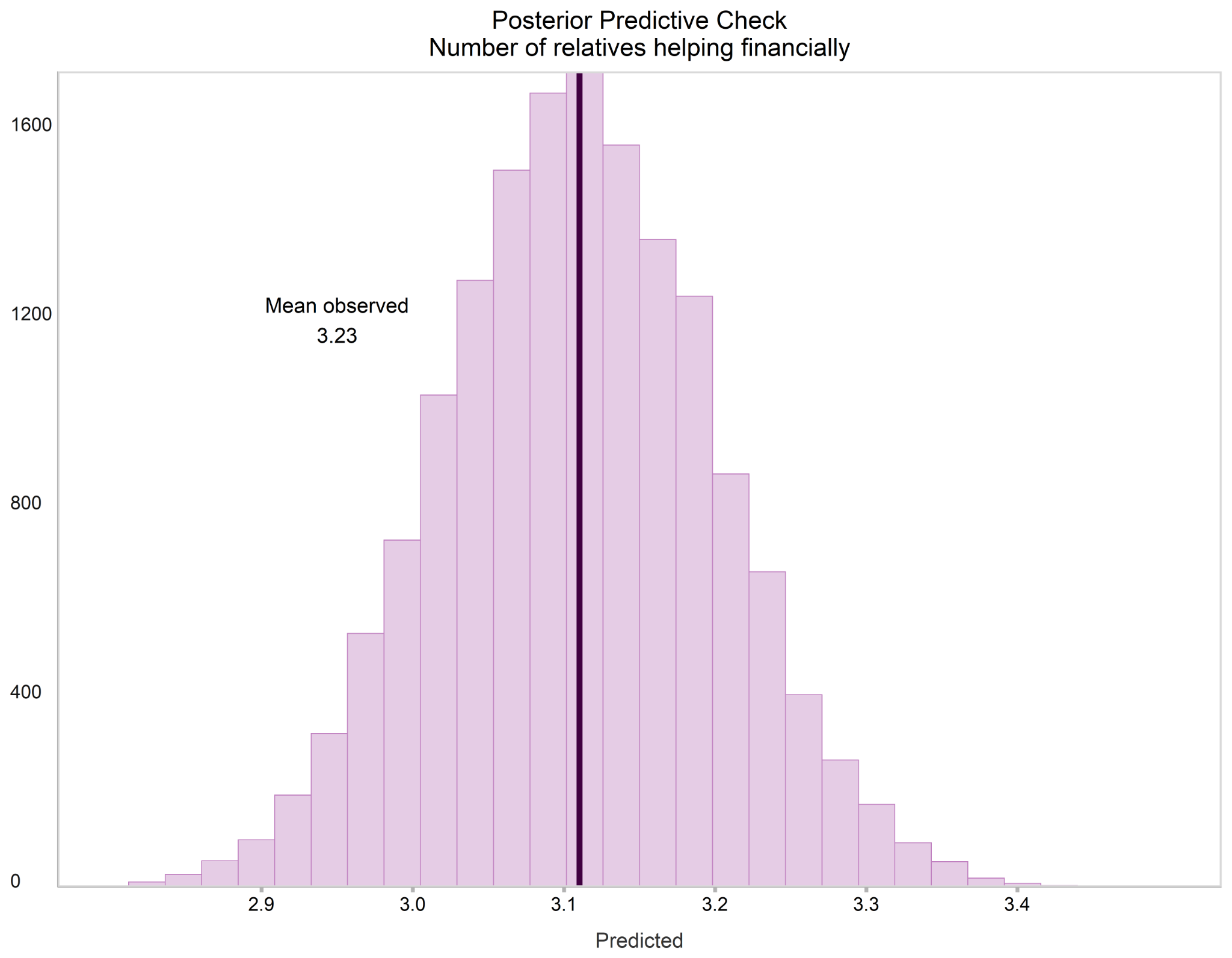


**Figure S9a** Posterior predictive check, Number of relatives providing financial support (PPC8)


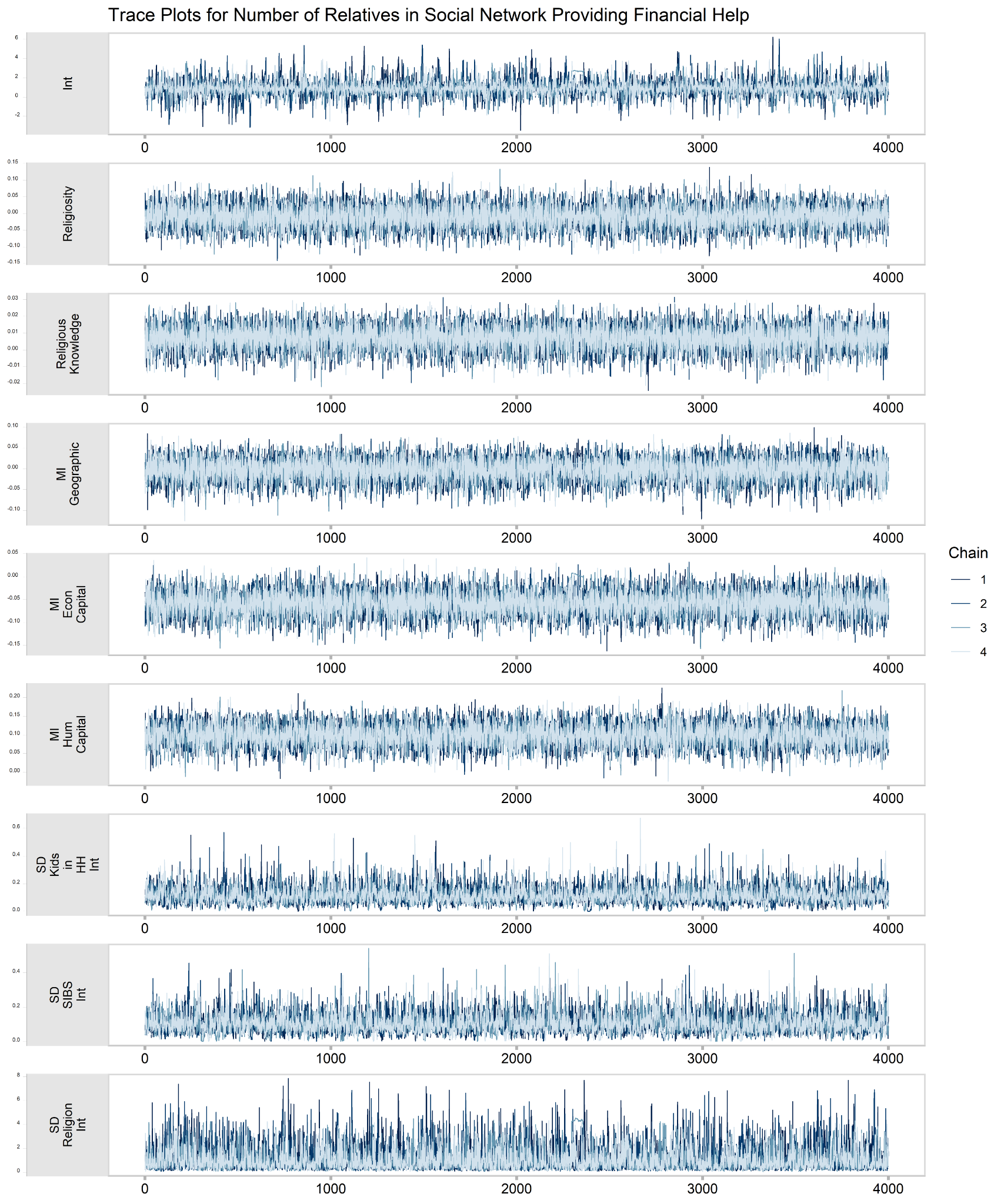


**Figure S9b** Markov Chains, Number of relatives providing financial support (Trace_plot_8)


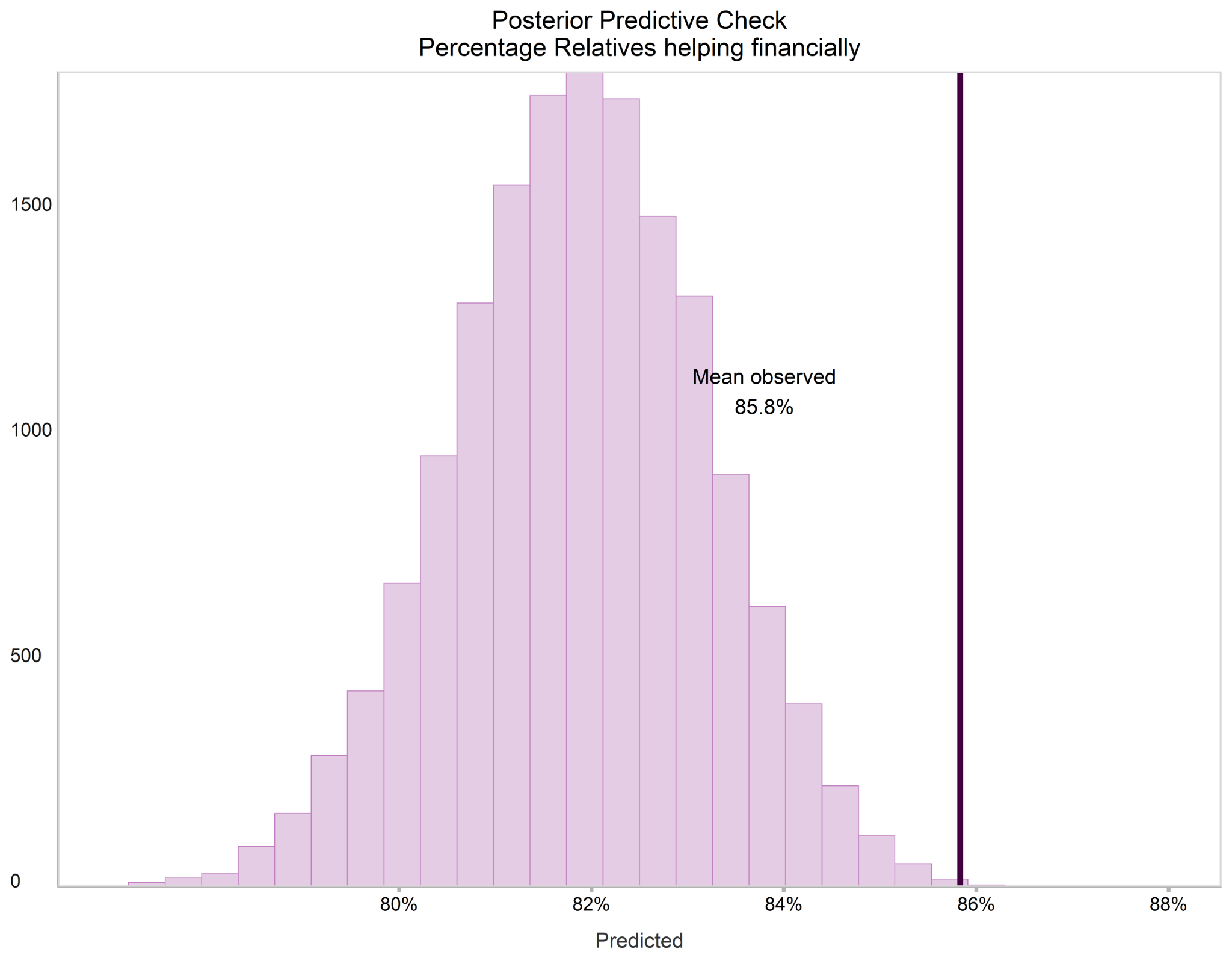


**Figure S10a** Posterior predictive check, Percentage of network helping financially that are relatives (PPC9)


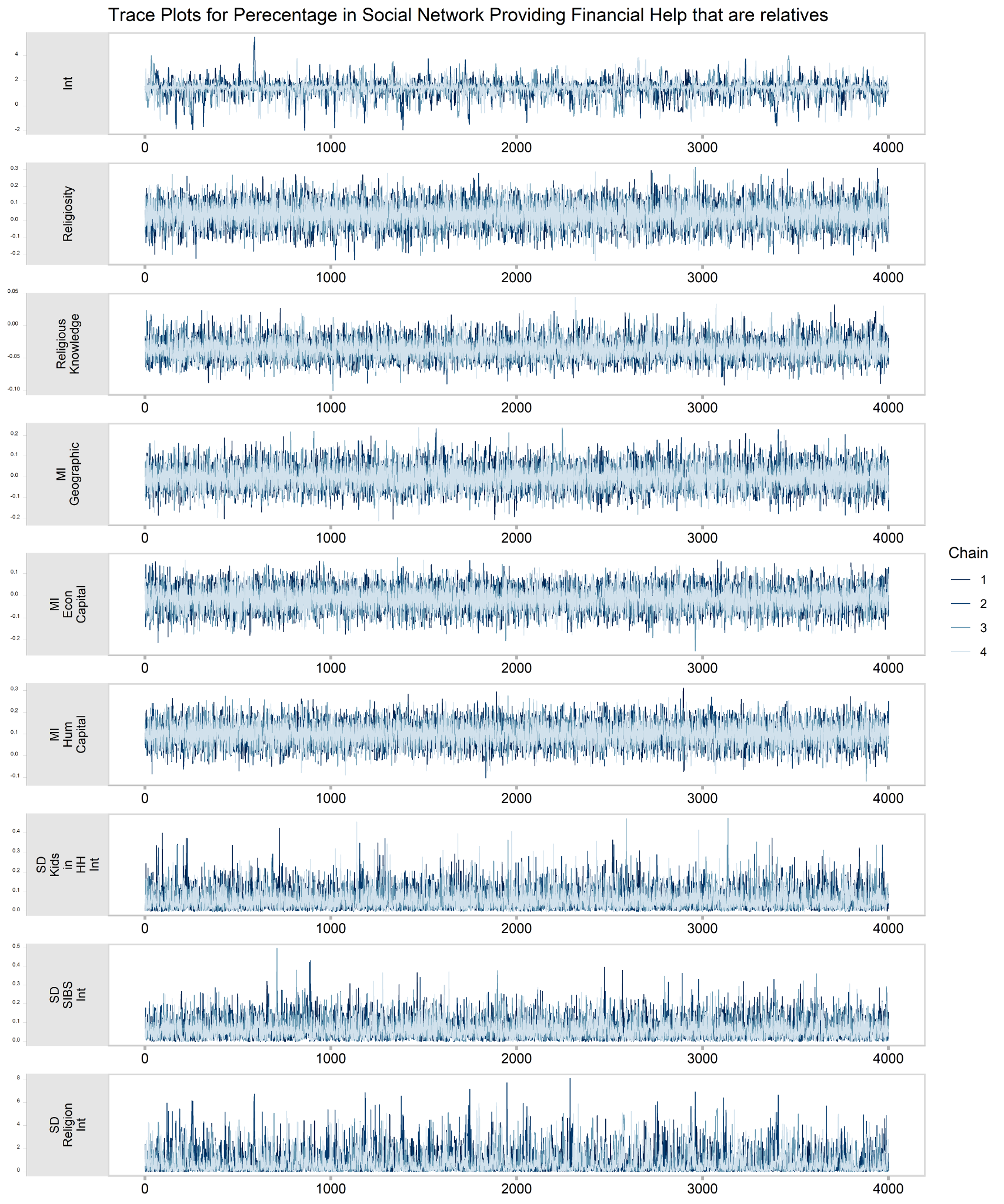


**Figure S10b** Markov Chains, Percentage of network helping financially that are relatives (Trace_plot_9)


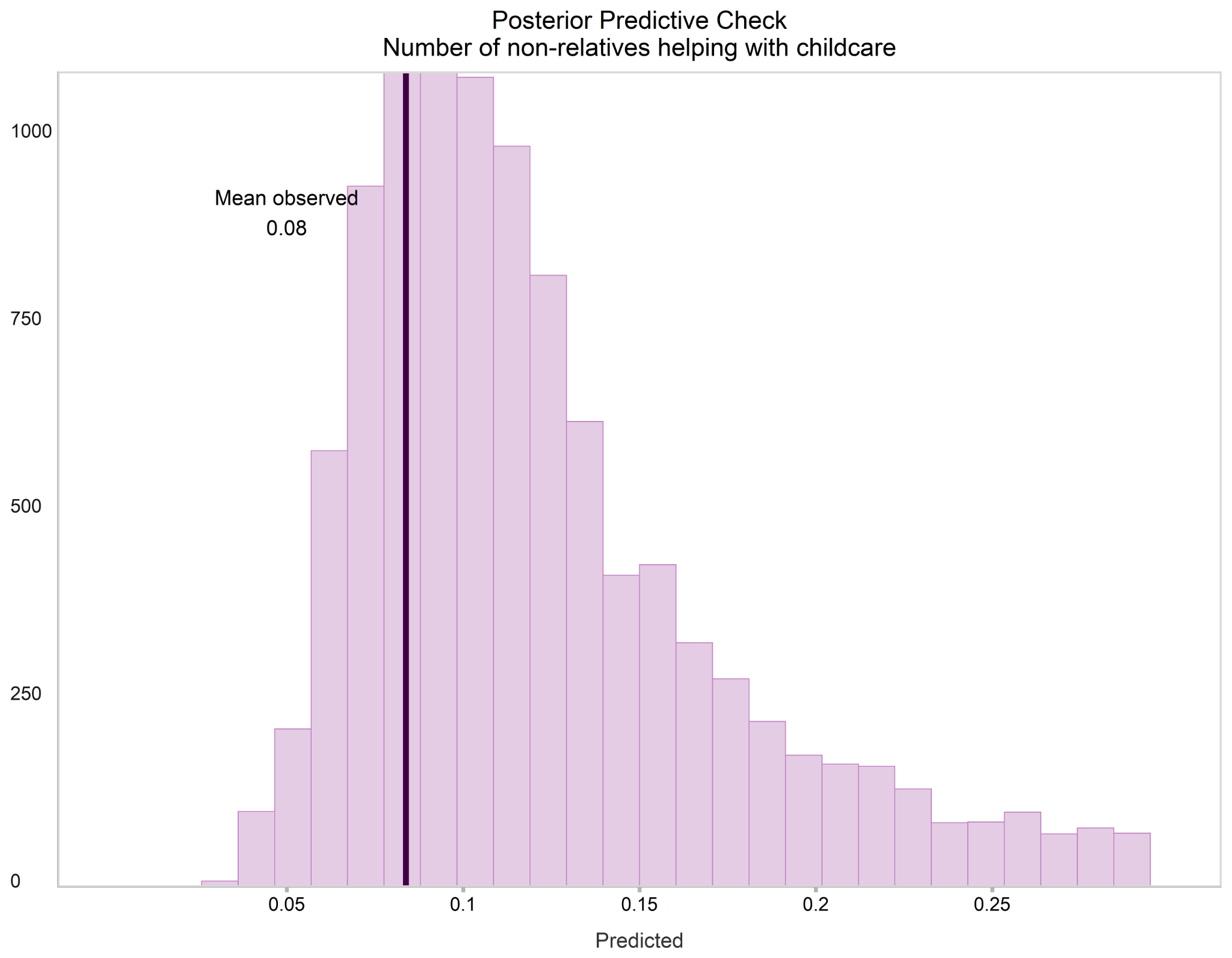


**Figure S11a** Posterior predictive check - Number of non-relatives helping with childcare


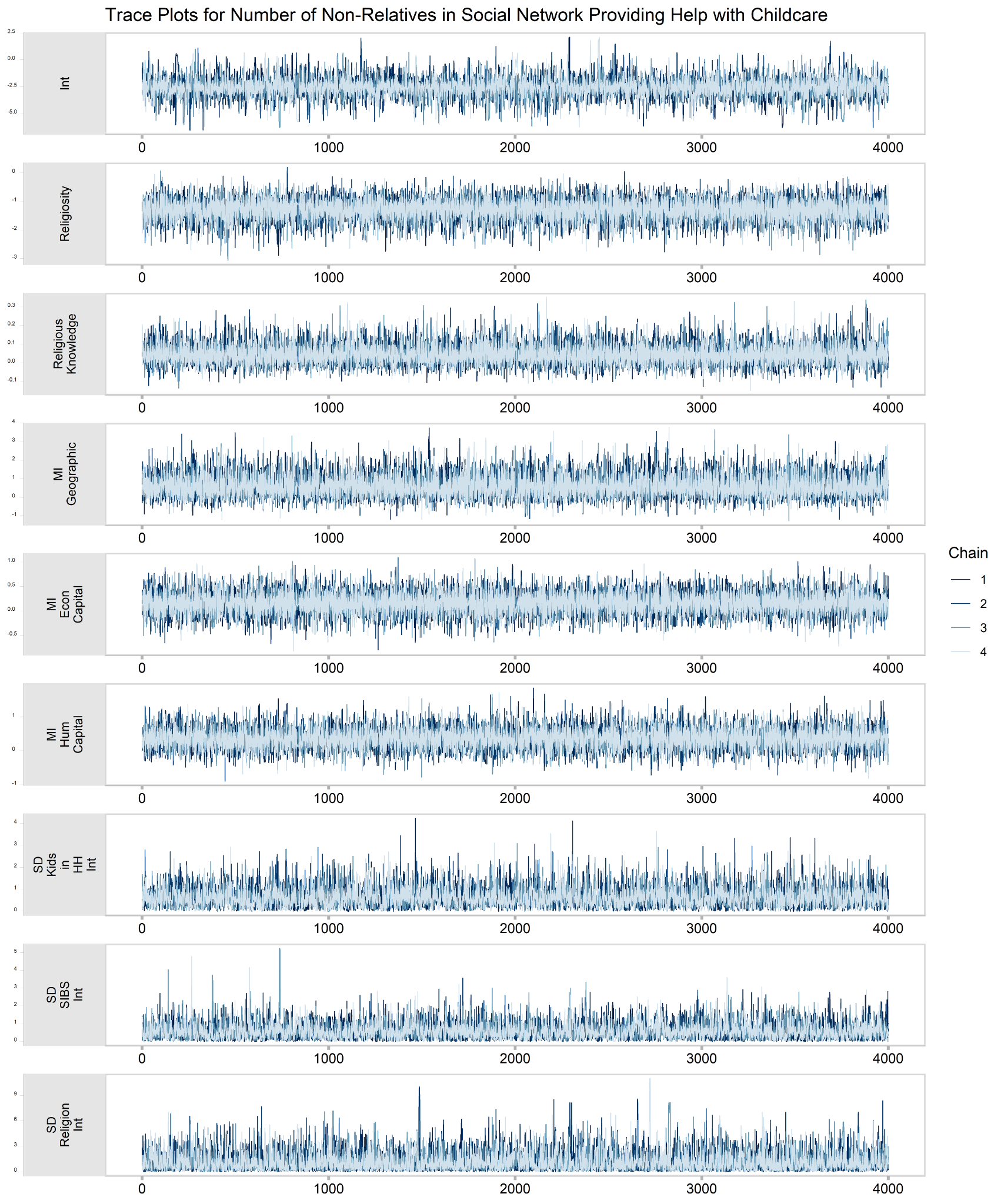


**Figure S11b** Markov Chains - Number of non-relatives helping with childcare


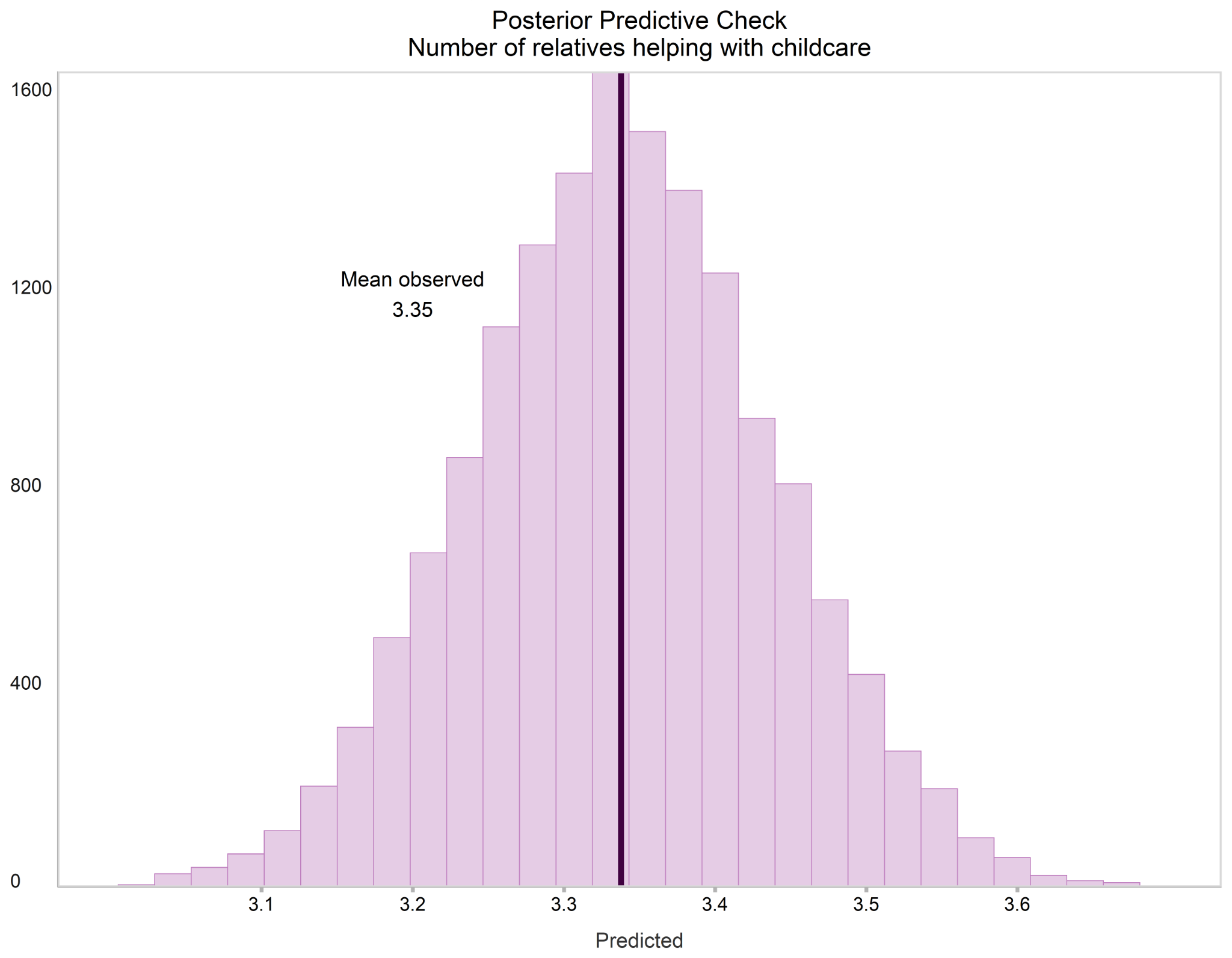


**Figure S12a** Posterior predictive check- Number of relatives helping with childcare


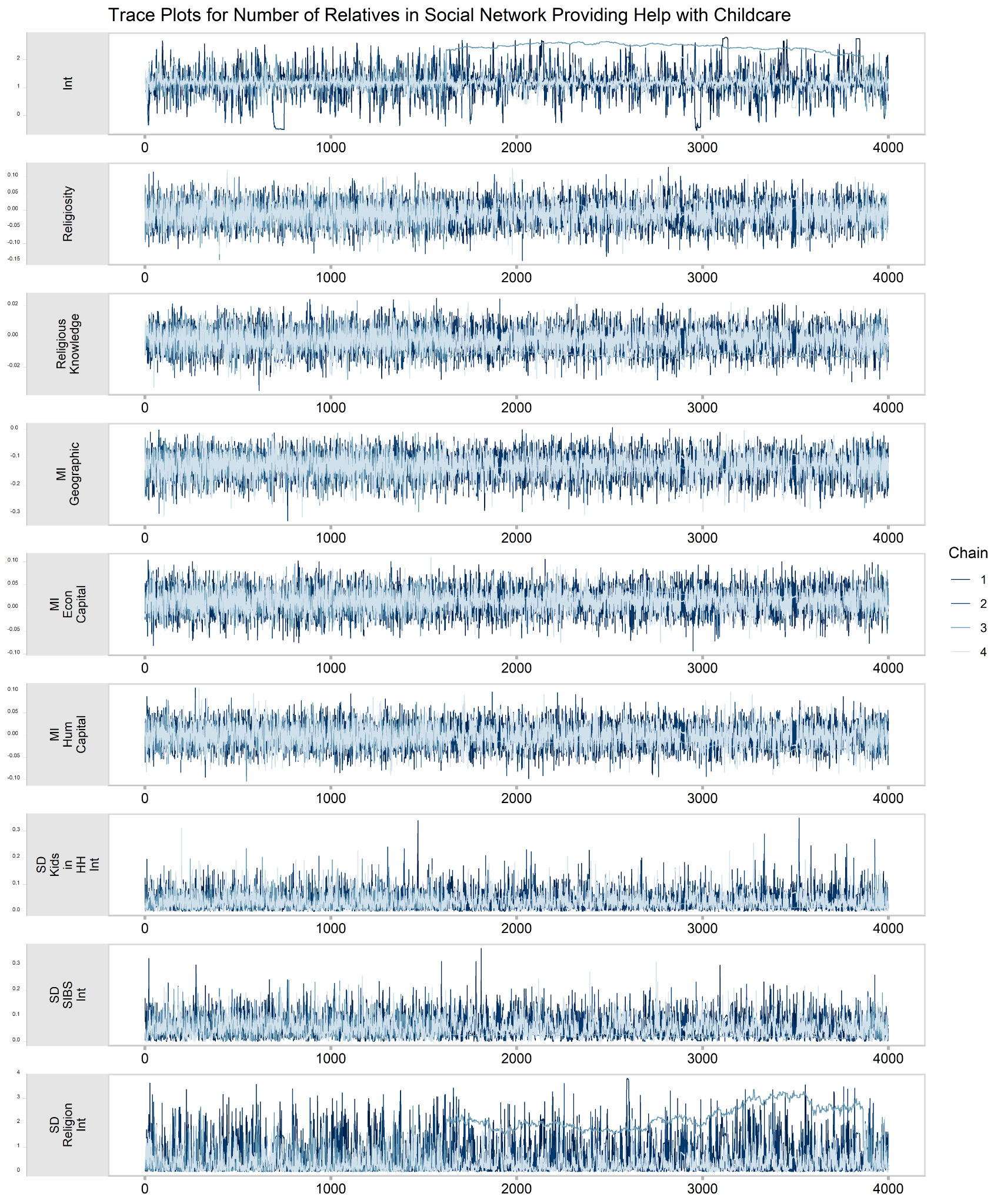


**Figure S12b** Markov Chains Number of relatives helping with childcare


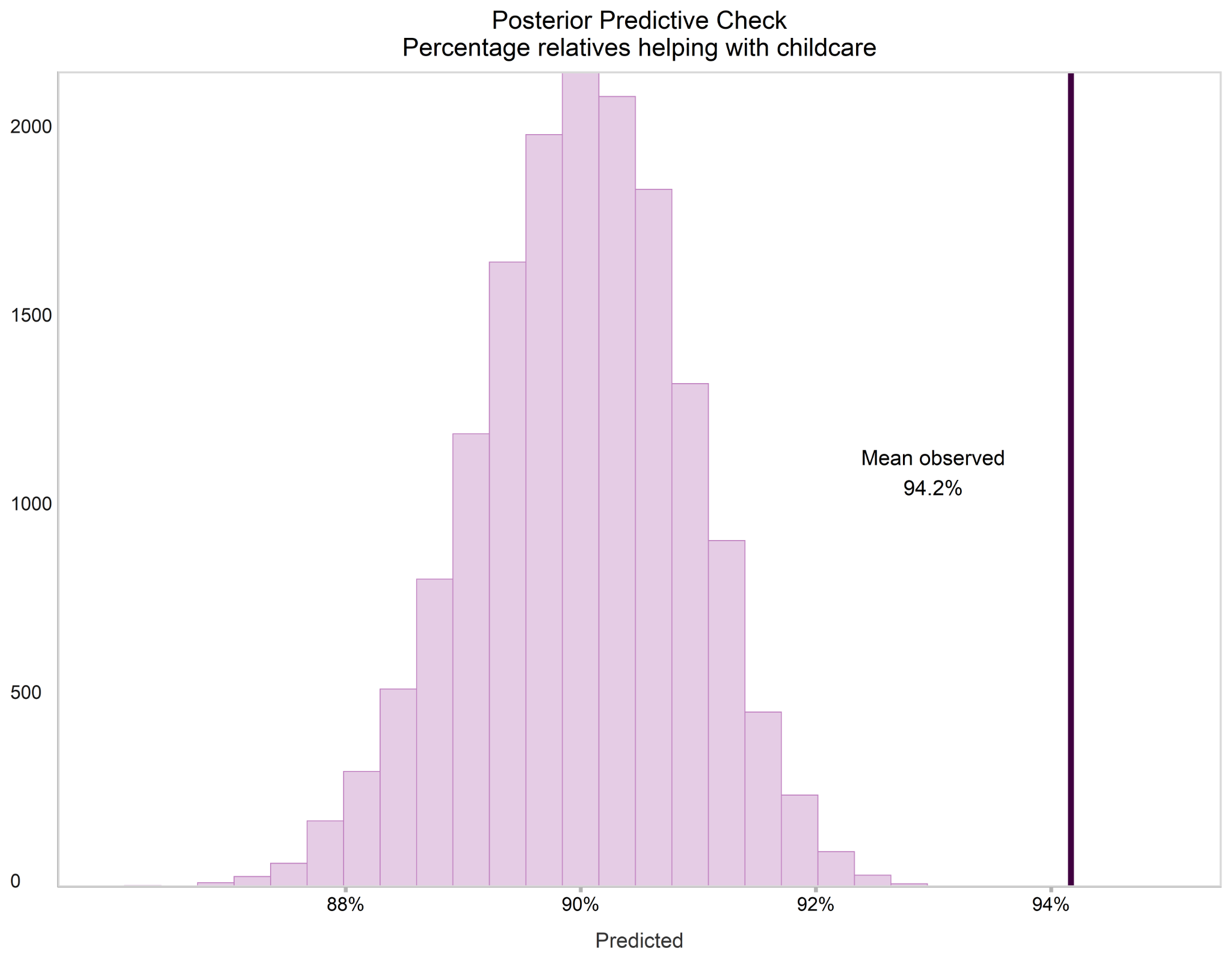


**Figure S13a** Posterior predictive check - Percentage of people in social network helping with childcare who are relatives.


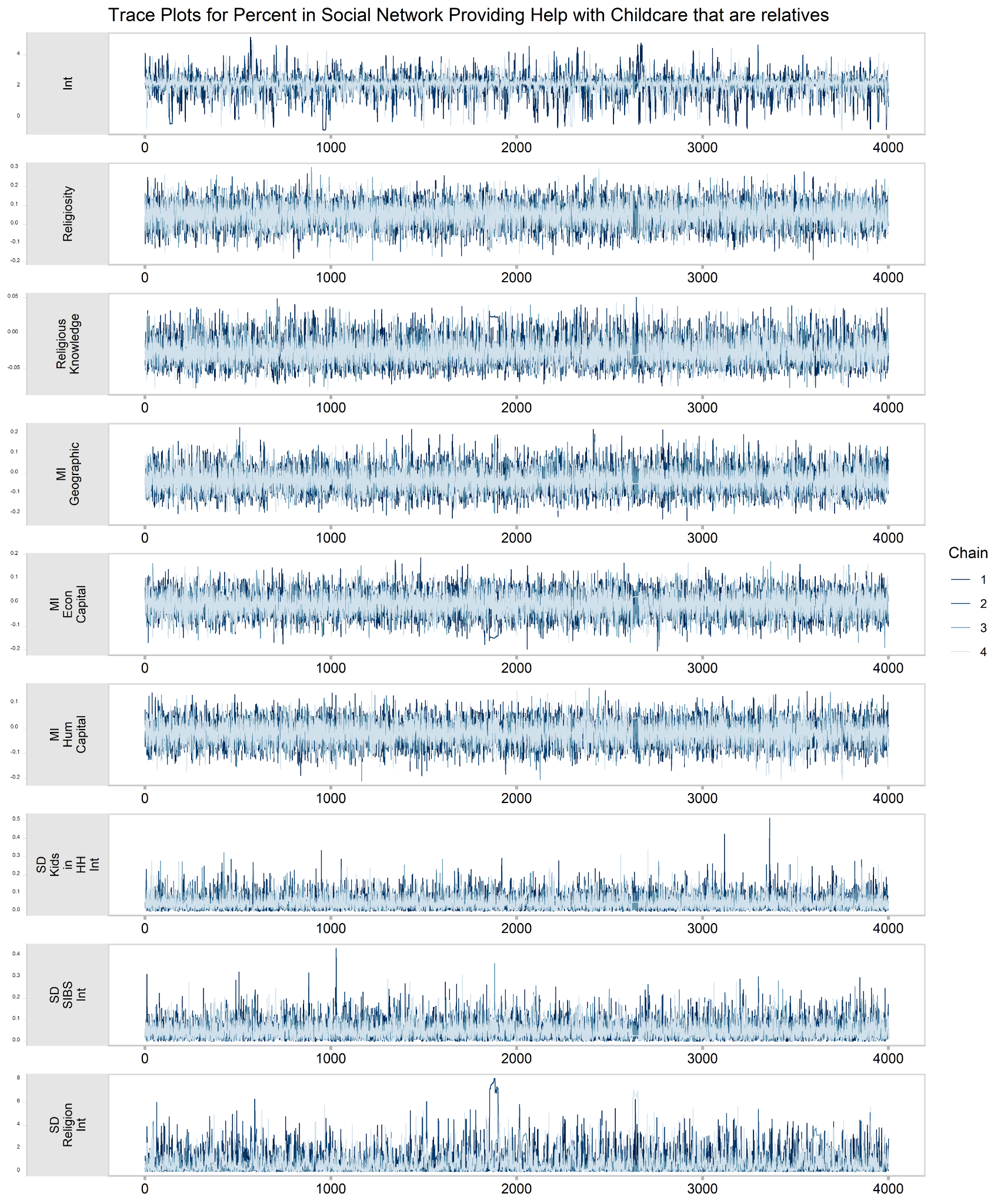


**Figure S13b** Markov Chains- Percentage of people in social network helping with childcare who are relatives.


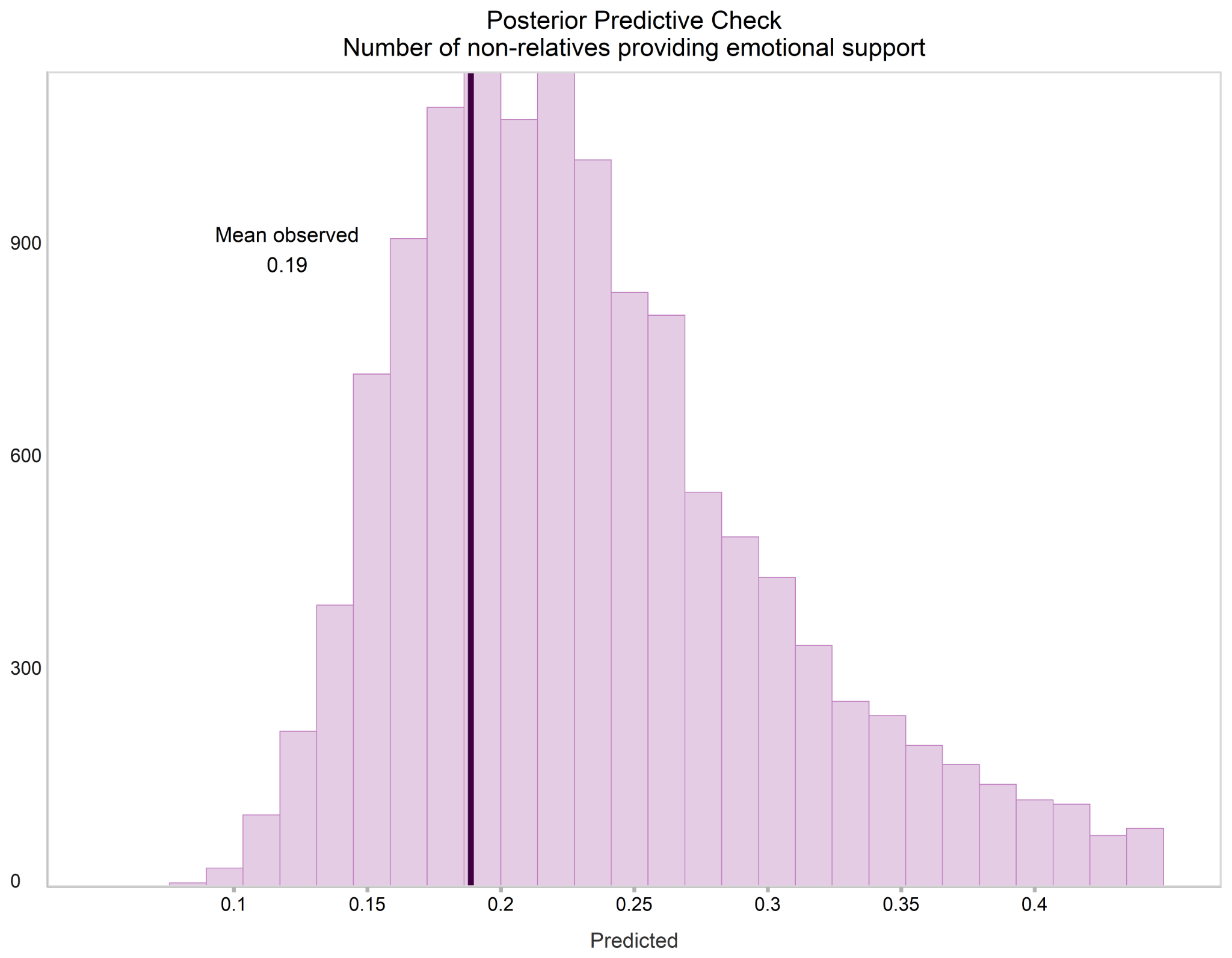


**Figure S14a** Posterior predictive check - Number of non-relatives providing emotional support.


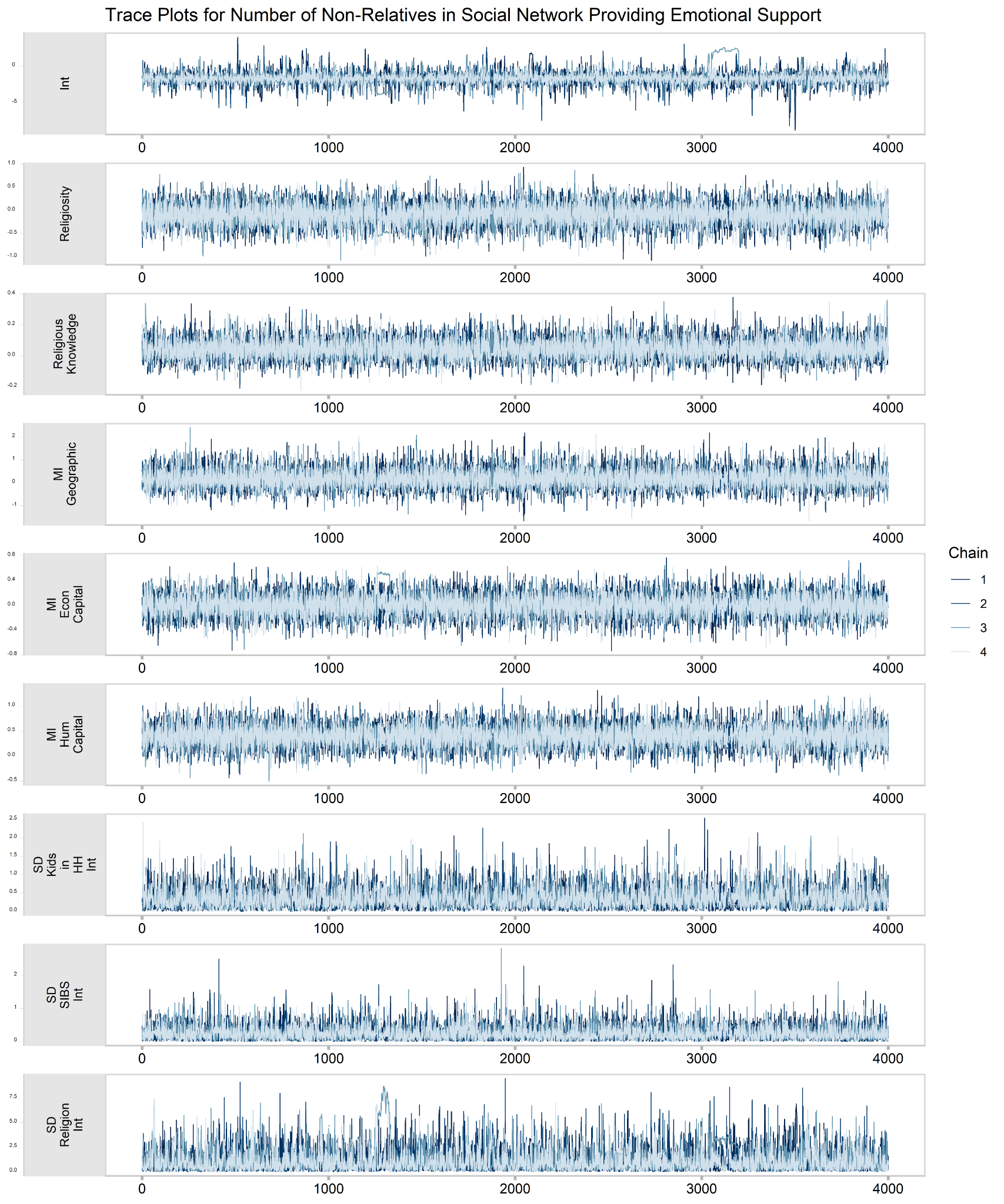


**Figure S14b** Markov Chains- Number of non-relatives providing emotional support.


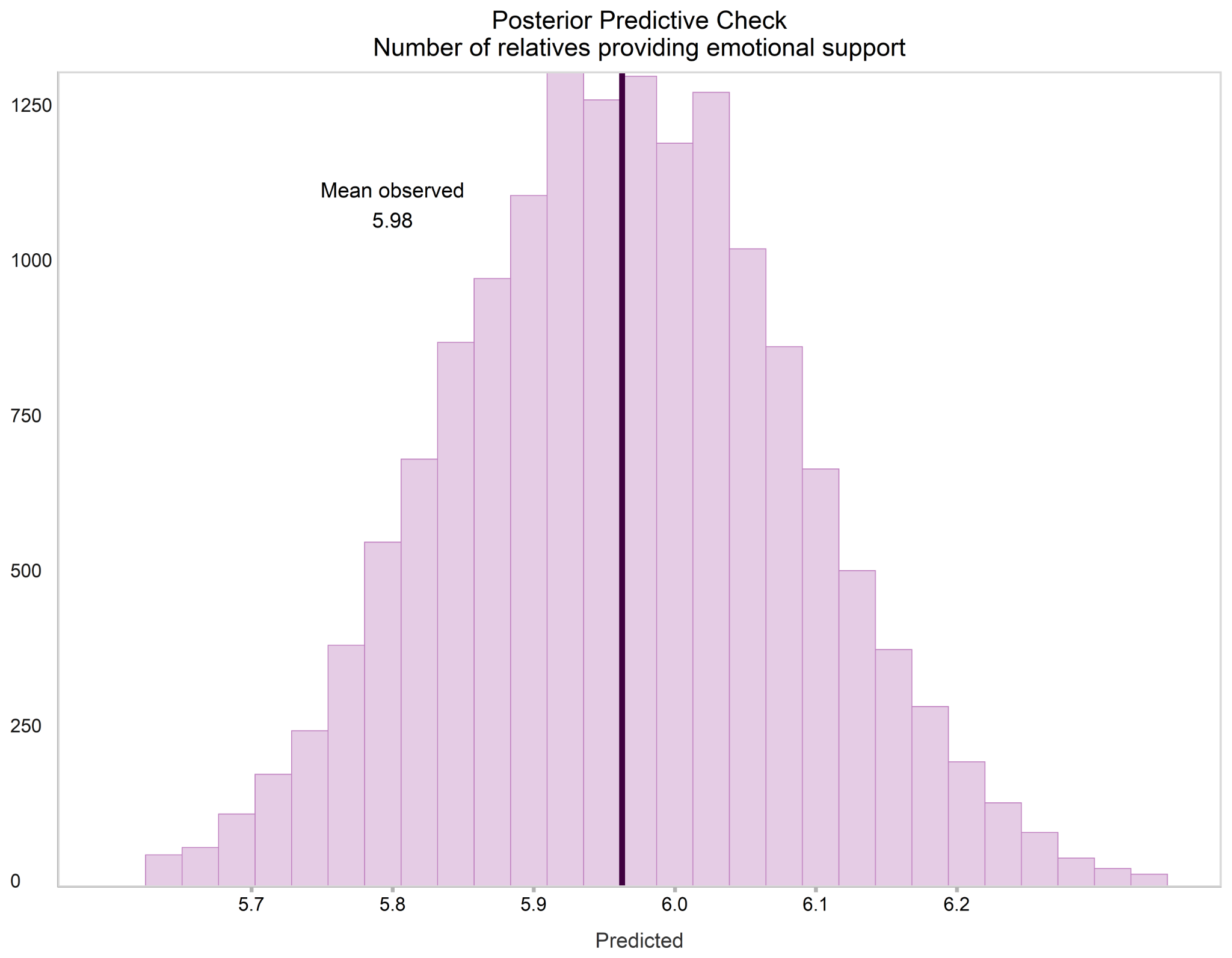


**Figure S15a** Posterior predictive check - Number of Relatives providing emotional support.(PPC14)


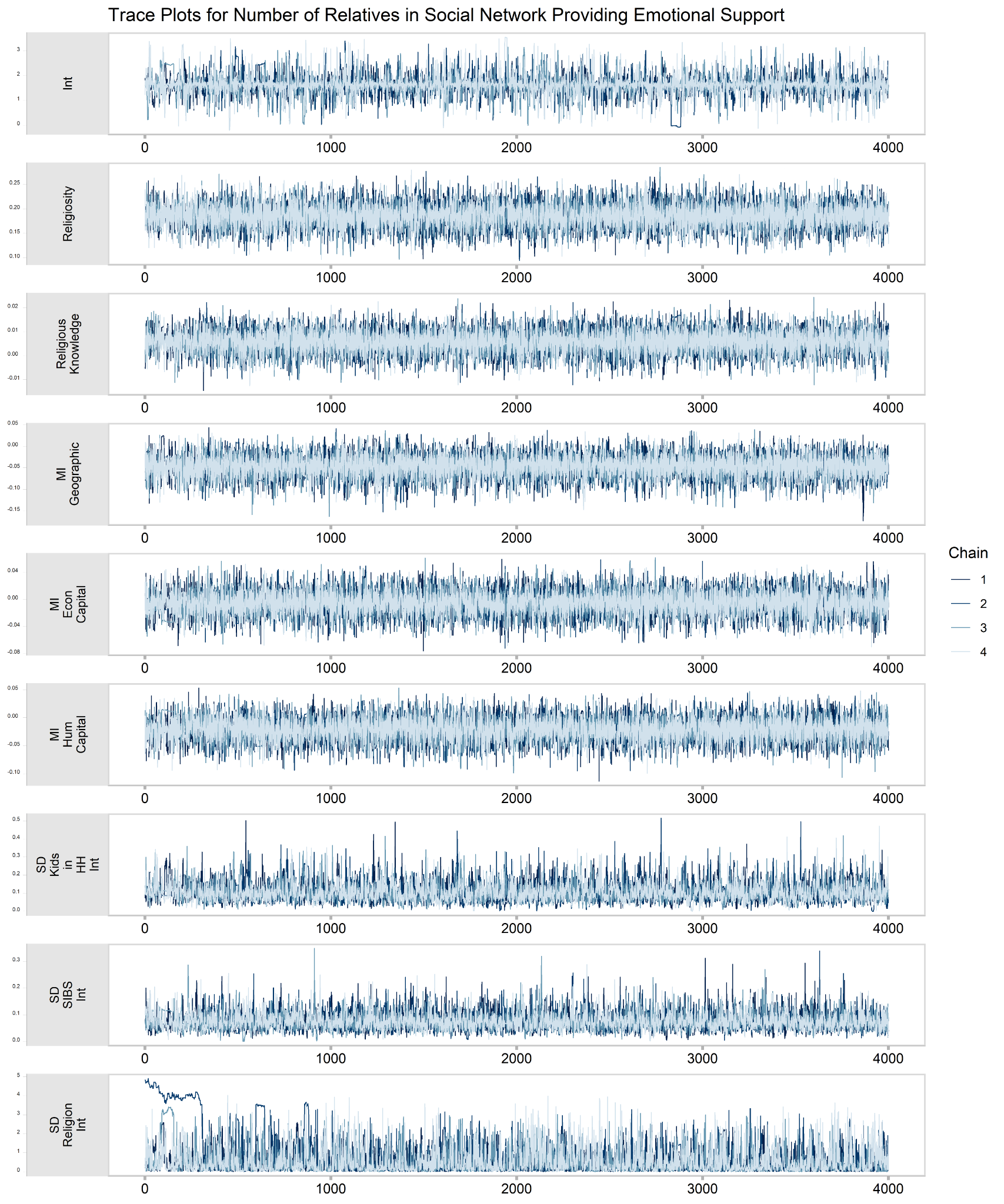


**Figure S15b** Markov Chains-Number of Relatives providing emotional (Trace_plot14)


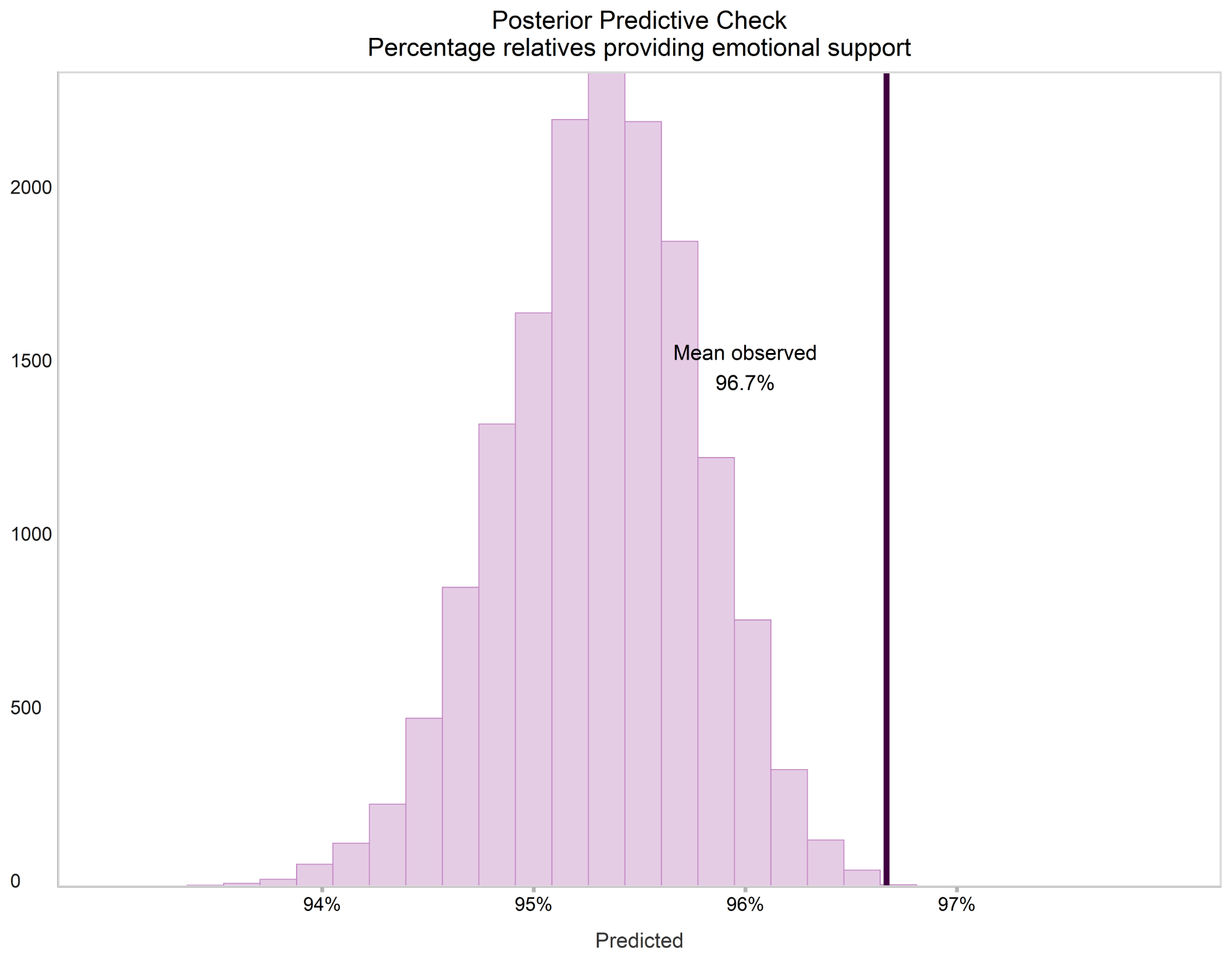


**Figure S16a** Posterior predictive check - Percentage of people in social network providing emotional support who are relatives (PPC15)


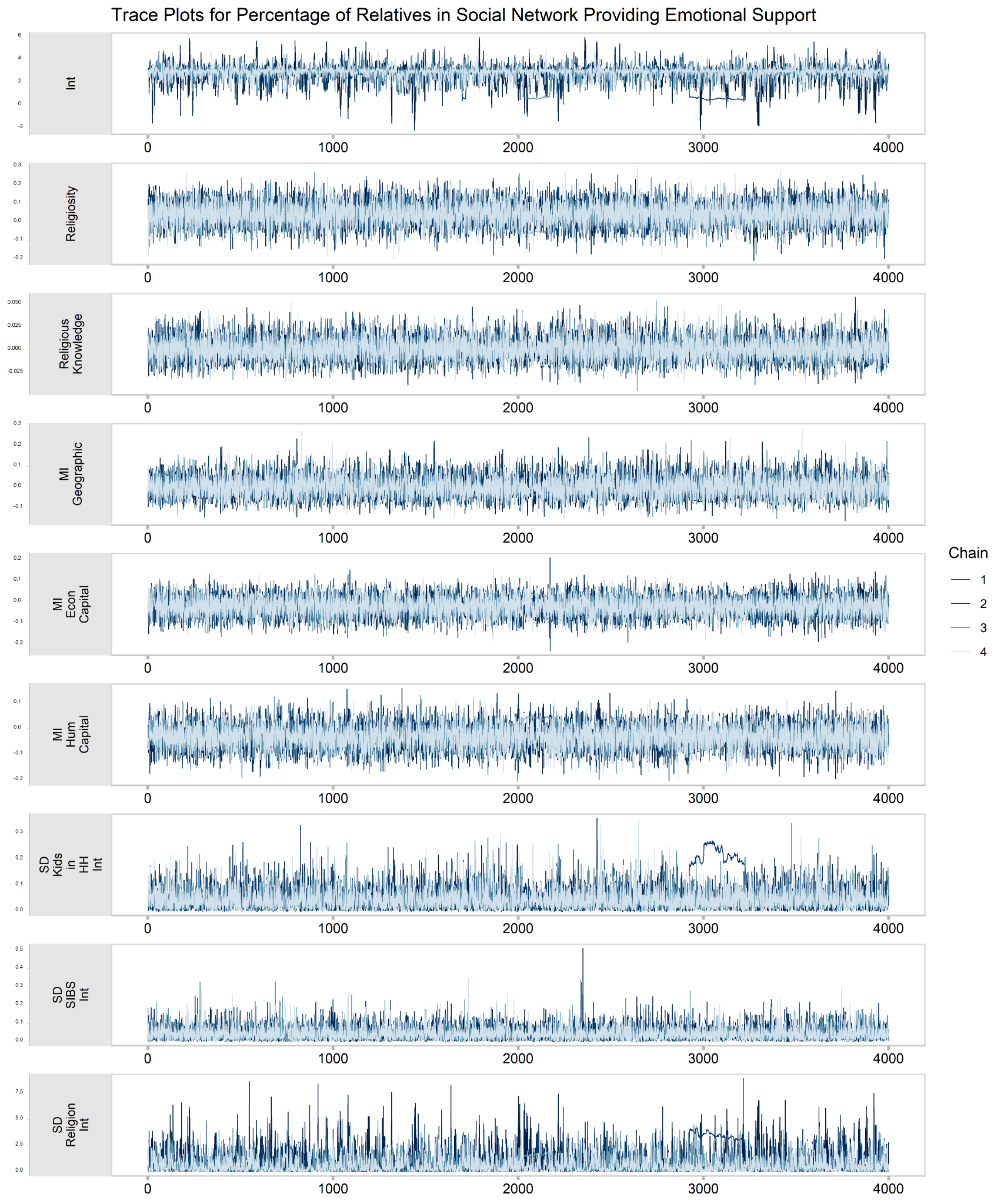


**Figure S16b** Markov chain convergence - Percentage of people in social network providing emotional support who are relatives (trace_plot 15)


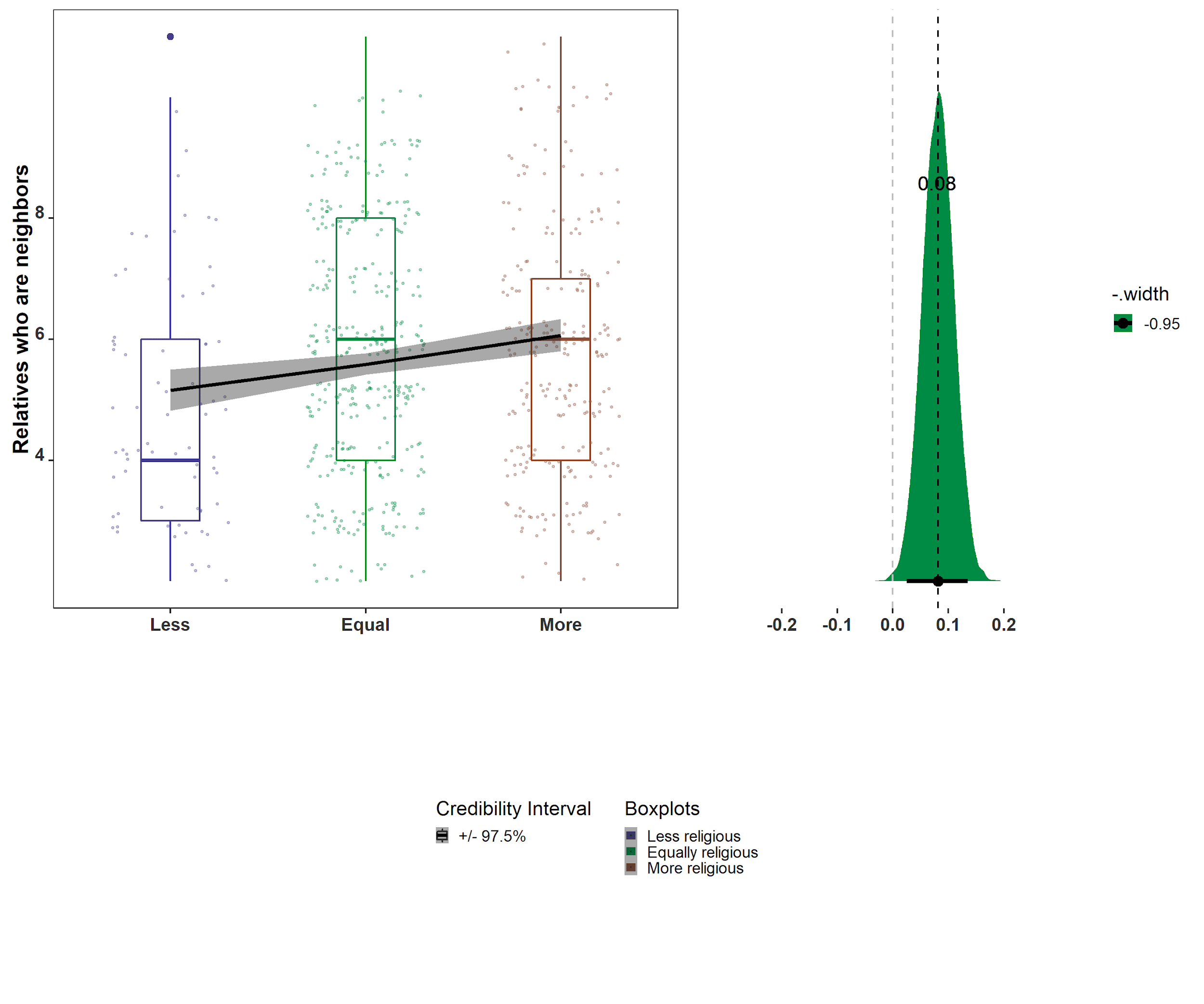


**Figure S17:** More religious individuals have more relatives in their social networks who are neighbors


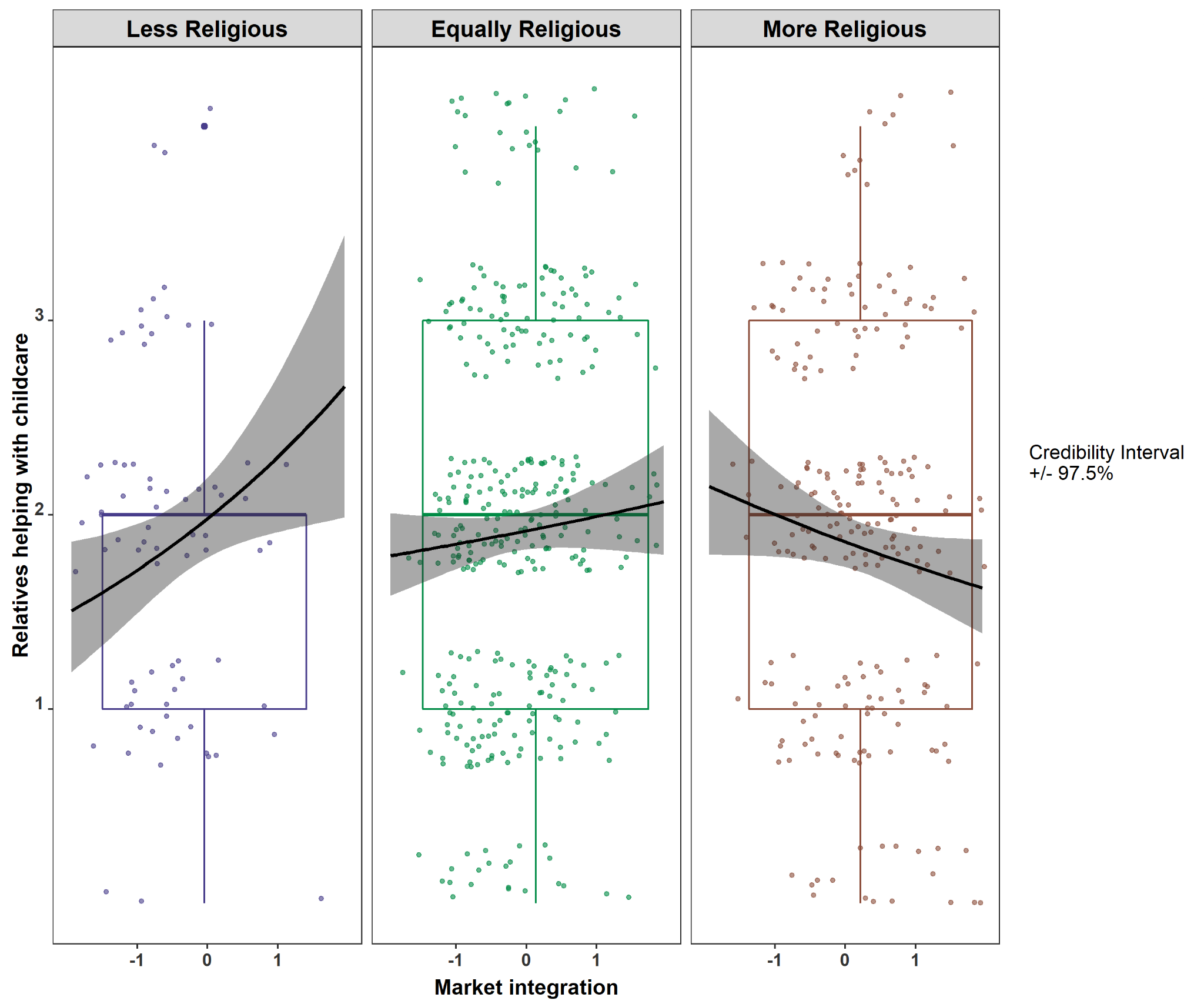


**Figure S18:** Less religious and more market integrated individuals have more relatives who help with childcare (left panel) and more religious individuals who are more integrated receive less help (right panel).
